# Circulating memory B-cell receptor repertoire analysis identifies novel candidate antibodies against metastatic melanoma in immunotherapy-responsive patients

**DOI:** 10.3389/fimmu.2025.1636722

**Published:** 2025-10-09

**Authors:** Fabio Nicolini, Anna Gaimari, Lucia Mazzotti, Anna De Lucia, Miriana Ghirelli, Valentina Ancarani, Patricia Borges de Souza, Matteo Zurlo, Luca Gazzola, Chiara Magnoni, Simona Capobianco, Davide Angeli, Sara Bravaccini, Claudio Cerchione, Massimo Guidoboni, Luisa Lanfrancone, Federica Marocchi, Roberta Maltoni, Maria Maddalena Tumedei, Francesco Limarzi, Luigi Pasini, Laura Ridolfi, Massimiliano Mazza

**Affiliations:** ^1^ Advanced Cellular Therapies and Rare Tumors Unit, Istituto di Ricovero e Cura a Carattere Scientifico (IRCCS) Istituto Romagnolo per lo Studio dei Tumori (IRST) “Dino Amadori”, Meldola, Italy; ^2^ Department of Biomedical and Neuromotor Sciences, Cell Signalling Laboratory, University of Bologna, Bologna, Italy; ^3^ Unit of Biostatistics and Clinical Trials, Istituto di Ricovero e Cura a Carattere Scientifico (IRCCS) Istituto Romagnolo per lo Studio dei Tumori (IRST) “Dino Amadori”, Meldola, Italy; ^4^ Department of Medicine and Surgery, University of Enna “Kore”, Enna, Italy; ^5^ Hematology Unit, Istituto di Ricovero e Cura a Carattere Scientifico (IRCCS) Istituto Romagnolo per lo Studio dei Tumori (IRST) “Dino Amadori”, Meldola, Italy; ^6^ Unità Operativa Complessa (UOC) Clinical Oncology, University Hospital of Ferrara, Ferrara, Italy; ^7^ Department of Experimental Oncology, European Institute of Oncology Istituto di Ricovero e Cura a Carattere Scientifico (IRCCS) (IEO), Milan, Italy; ^8^ Healthcare Administration, Istituto di Ricovero e Cura a Carattere Scientifico (IRCCS) Istituto Romagnolo per lo Studio dei Tumori (IRST) “Dino Amadori”, Meldola, Italy; ^9^ Biosciences Laboratory, IRCCS Istituto Romagnolo per lo Studio dei Tumori (IRST) “Dino Amadori”, Meldola, Italy; ^10^ Pathology Unit, Morgagni-Pierantoni Hospital, Azienda Unità Sanitaria Locale (AUSL) Romagna, Forlì, Italy

**Keywords:** metastatic melanoma, immunotherapy, responders, B-cell receptor repertoire, patient-derived antibodies, immune checkpoint inhibitors

## Abstract

**Background:**

By examining the B-cell receptor (BCR) repertoire of metastatic melanoma (MM) patients with favorable treatment outcomes, it is now possible to identify unique patterns of immune responses, with the potential of discovering novel antitumor antibodies.

**Methods:**

Here, we isolated CD27-positive circulating memory B cells from non responders, partial responders, and complete responders MM patients to first-line therapy with anti-PD-1 immune checkpoint inhibitor (ICI) nivolumab, to perform a BCR repertoire sequencing analysis. We looked for complementarity-determining region 3 (CDR3) sequences that were enriched (*de novo* formed) following ICI treatment. Fully-human immunoglobulins were then produced in Expi293F™ cells using CDR3-sequencing information and tested for specificity and sensitivity on different MM cell lines and patient-derived xenograft cells by flow cytometry and by immunohistochemistry on human tissue microarrays.

**Results:**

As a result of immunotherapy stimulation in responder patients, we observed that some CDR3 clonotypes have emerged *de novo*. Among the nine candidate antibodies we assessed, two antibodies exhibited encouraging tumor-targeting properties, although they also showed a degree of cross-reactivity with normal skin and melanocytes.

**Conclusions:**

Although our study is based on a limited number of individuals, our observations indicate that it may be possible to further investigate the human response to immunotherapy for the identification of rare mature B clonotypes targeting plasma membrane antigens on tumor cells. These preliminary findings could contribute to the future development of fully human-compatible immunotherapies, pending additional validation and *in vivo* studies.

## Introduction

The approval of immunotherapy with immune-checkpoint inhibitors (ICIs) has reshaped the treatment of patients with metastatic melanoma (MM) over the past 13 years, bringing overall patient survival up to 60% (1, 2). Still, there remains a 30% subset of patients who undergo progression within a half-year timeframe, even when the targeting of the cytotoxic T-lymphocyte-associated protein 4 (CTLA-4), the programmed cell death protein 1 (PD-1) are combined (1, 2), and the programmed death-ligand 1 (PD-L1). This data emphasize the notion that inducing immune activation just by blocking the immune-suppressive streak of the tumor is not enough to hold back progression. Furthermore, the demand for improved therapeutic options comes from the evidence that inhibition of these immune checkpoints also inevitably increases the incidence of autoimmune toxicities (3). A peculiarity that is shared by all ICIs, whether they target CTLA4, PD-1, or PD-L1, is their ability to induce, in some cases, a durable objective response and absence of the frequent resistance mechanisms associated with conventional targeted therapies (4).

Efficacy and timing, as well as the duration of the ICI response for MM, are heterogeneous. However, those patients with tumors associated with a higher baseline response rate and enhanced mutational landscape (conceptually linked to the antigenic potential of a tumor) tend to have more extended positive outcomes (5). In the context of the so-called “cancer-immune set point” of a patient, clinical studies now aim to define the intrinsic relationship between tumor mutational burden, tumor microenvironment, tumor-infiltrating immune cells, circulating immune effectors and host factors to understand the variability in the clinical response of patients and to deliver more efficient immunological treatments (6).

Nowadays, immunotherapy success against tumors has been widely associated with a T cell specific response and immunosuppressive environment rewiring. However, there is growing evidence that tumor-infiltrating B cells and antibody-secreting plasma cells, collectively referred to as tumor-infiltrating B lymphocytes (TIL-Bs), also have a crucial and synergistic involvement in controlling tumor growth (7, 8). B cells may still have a divergent role, however, in modulating the immune response within the tumor microenvironment and the metabolic homeostasis by either inhibiting tumor development through antigen-fostered clonal expansion and production of tumor-reactive antibodies, which can be further retrieved in the bloodstream, or promoting tumor cell proliferation through the production of autoantibodies and tumor growth factors (9). Overall, B-cell immunotherapy has promising prospects for treating tumor metastasis. Nevertheless, B-cell-derived antibodies are recognized as pivotal to potentiate further the immune response of cytotoxic leukocytes and complement-mediated killing of cancer cells (10).

In the delicate balance between so many factors that either promote or suppress the immune response, the question arises of whether tumor-infiltrating plasma cells or circulating memory B cells are the main driving force for antitumor immunity. In melanoma, tumor-associated B cells are essential to sustain adequate T cell activation and trigger the inflammatory response with anti-PD-1 blockade, both in mice and in humans, with the number of peripheral-blood memory B cells detected during pretherapy being predictive of response and overall survival to ICI treatment (11, 12). Human memory B cells (typically defined as class-switched CD27+ B cells) are the most significant subset of naive-originated B cells in peripheral blood, and they play a critical role in establishing immune memory protection against repeated antigen exposure (13). Besides being mediators of adaptive humoral immunity, circulating memory B cells are also known to be involved in the recruitment of dendritic cells (DCs) to the site of tumor metastasis, enhancing the DC antigen-presentation role (14). B cells isolated from the peripheral blood of cancer patients directly induce apoptosis of tumor cells *in vitro* (15). Further studies show that switched memory B cells are the primary subtype of proliferating TIL-Bs contributing to the formation of tertiary lymphoid structures (TLSs) at chronically inflamed sites (16), such as tumors, during immune checkpoint blockade therapy (17).

Sequencing analysis of the diversity of the B-cell receptor (BCR) repertoire expressed by an individual patient’s total B cell population has scaled up the identification of new potential target antigens for practical evaluation of immune response in cancer (18, 19). Affinity maturation of a specific BCR occurs in the germinal centers associated with TLSs by somatic hypermutation (SHM), and it is crucial to determine receptor-mediated selection that triggers efficient anti-cancer mechanisms (20). However, the clinical value of BCR repertoire analysis from peripheral blood memory B cells, as an accessible blood-based tool for intercepting the anti-tumor humoral response initiated *in situ* and predicting ICI treatment efficacy in MM, remains unexplored.

Over-ten-year follow-up studies, including neoadjuvant trials with single-agent PD-1 blockade, finally reveal the prospect of long-term outcomes in advanced staged melanoma following ICI treatments (21, 22). Interestingly, enrichment of switched memory B cells (those that have transitioned into plasma cells) in the TLSs of metastatic tumors is associated with prolonged survival (23). Also, patients with an increased frequency of switched memory B-cells are more likely to benefit from ICI treatment, as indicated by the relative enrichment of B cells in the blood of responders and non-responders (24). B cells are necessary for generating optimal inflammatory anti-melanoma T lymphocyte activation after immunotherapy response, where specific subtypes of B cells can predict the clinical outcome (11, 12).

To further explore the contribution of human memory B cells in ICI treated MM patients and the existence of targeting antibodies as a long-term anti-tumor humoral response, we developed a screening strategy based on BCR repertoire analysis by next generation sequencing (NGS) coupled with *ex vivo* production of tumor-reactive and tumor-specific antibodies.

## Materials and methods

### Sample collection and isolation of peripheral blood memory B-cells

Blood samples (50 mL) from six metastatic melanoma patients were collected into EDTA-containing Vacutainer tubes, both at the time of diagnosis (Pre) and after (Post) first-line nivolumab treatment (3 mg/kg), as detailed in **Table 1**. The selected samples belong to patients who maintained a consistent clinical response throughout the entire treatment and were collected close to the time of clinical reassessment by the physician. PBMC isolation was performed using the Leucosep method (Greiner Bio-One). First, Leucosep tubes were filled with 13 mL of separation media and centrifuged at 250 x g for one minute. Then, 50 mL of blood were diluted (1:1) with Hanks′ Balanced Salt Solution (HBSS), carefully layered on the porous membrane, and centrifuged at 620 × g for 10 min without brakes at room temperature. After centrifugation, the lymphocyte layer between the plasma/HBSS and the membrane transferred into a clean tube, and washed with HBSS. Samples were then centrifuged at 620 × g for 10 min, the supernatant was removed, and 10 mL of fresh HBSS was added to wash the cells. The resulting pellet was resuspended in 2 mL of ACK Lysing buffer for 2–5 minutes to lyse red blood cells, and then washed with HBSS twice. PBMCs were counted with trypan blue dye exclusion test to determine the number of viable cells and then resuspended in 10% DMSO-containing freezing solution to be stored in liquid nitrogen. Memory B-cells were isolated from the PBMCs by using a memory B-cell isolation kit (Miltenyi, 130-093-546) following manufacturer instructions. Isolated memory B-cells were subsequently divided in two replicate tubes (called replicate 1 and 2), centrifuged, and resuspended in LTR buffer (Qiagen) to assure RNA stability.

**Table 1:** Clinical information and data collection for PBMCs and memory B cells isolated from melanoma patients.

Patient ID	Diagnosis	Therapy	Clinical response	Sample collection (months after treatment start)	PBMCs PRE (*10^6)	Memory B cells PRE (*10^6)	PBMCs POST (*10^6)	Memory B cells POST (*10^6)
1	Melanoma	First-line Nivolumab	PD	13,0	15,0	0,015	13,8	0,010
4	Choroidal melanoma	First-line Nivolumab	PD	7,0	20,0	0,017	26,0	0,0128
7	Nodular melanoma	First-line Nivolumab	PR	15,0	21,6	0,014	15,6	0,004
9	Lentigo maligna melanoma	First-line Nivolumab	PR	10,0	15,4	0,030	20,0	0,020
3	Lentigo maligna melanoma	First-line Nivolumab	CR	11,0	14,7	0,046	15,4	0,040
8	Nodular melanoma	First-line Nivolumab	CR	7,0	15,9	0,013	22,8	0,011

Six melanoma patients who responded or did not respond to first-line anti-PD-1 therapy were included in the B-cell receptor repertoire analysis (PD, progressive disease; PR, partial response; CR, complete response). Peripheral blood mononuclear cells (PBMCs) and memory B cells were collected pre- and post-therapy from peripheral blood (number of cells per ml).

The study was performed following the principles of the Declaration of Helsinki. All patients (or that person’s parent or legal guardian) in any type of qualitative or quantitative research, provided written informed consent to participate in the research. The study was approved by the Scientific and local Ethics Committee (Comitato Etico della Romagna, C.E.Rom.) of IRST-IRCCS (Prot. N. 5115/5.3 July 4th, 2017, and Prot. N. 3534/51/2018 May 10th, 2018, respectively).

### RNA extraction and sequencing

RNA was purified using Qiazol solution (Qiagen) according to the manufacturer’s protocol. Preparation of the 5’RACE cDNA libraries was performed as previously described (25, 26). Appropriate oligos were used for the immunoglobulin G (IgG) specific CDR3 (IgG CDR3) or the immunoglobulin H (IgH) isotype-independent (IgH CDR3) CDR3 sequencing. Sequencing of CDR3 regions was performed on Illumina MiSeq with a 150bp paired end protocol. Sequencing of the full-length IgG (entire variable heavy, VH, chain) was performed on Illumina MiSeq with a 300bp paired end protocol. Immune repertoires were extracted using MiXCR (MiLaboratories Inc, USA) (27) a software platform for analysis of Next-Generation Sequencing (NGS) data for immune profiling.

### B-cell repertoire sequence analysis

From the full length IgG sequencing, a very limited number of reads have been obtained and a very low level of overlapping among the two replicates was observed. Consequently, to select the candidate clonotypes we relied only on CDR3 sequences from IgG CDR3 and IgH CDR3 sequencing, for which a higher number of reads was available (**Supplementary Tables 1**, **2**). However, the full length IgG sequencing was used to obtain the complete VH sequence, FR1-CDR1-FR2-CDR2-FR3-CDR3-FR4, for each selected clonotype. Analysis and graphical processing of the data generated by MiXCR were conducted with the VDJviz software, as previously described (28). The CDR3 sequences from all samples were ranked according to the number of read counts and clone fractions. For each DNA sequence detected, VDJviz also takes into consideration the amino acid sequences along with the V-D–J segment information. Basic sequencing data, such as reads, diversity, mean clonotype frequency, and mean CDR3 nucleotide length, are reported in **Supplementary Tables 1**, **2**. Due to the nature of our sequencing data, which allowed identification of the heavy chain CDR3 or full heavy chain sequence along with isotype but did not provide paired native light chain sequences, we were unable to clone the native light chains.

### Antibody production

A total of nine CDR3 sequences were selected and used to produce the candidate antibodies. From the IgG CDR3 sequencing we identified 7 potential antibodies that we called A-G, while from the IgH CDR3 sequencing we identified and generated antibodies H and I. The candidate antibodies were produced in a fully-human immunoglobulin-G format (human/hIgG1 human/hkappa) by co-transfecting Expi293F cells (Thermo Fisher Scientific, cat. n.A14527) with a specific heavy chain (HC) and a common random k-light chain (LC) sub-cloned in pcDNA3.4-expressing vectors. Expi293F cells were grown in serum-free Expi293F™ Expression Medium (Thermo Fisher Scientific). The cells were maintained in Erlenmeyer Flasks (Corning Inc.) at 37°C with 8% CO2 on an orbital shaker (VWR Scientific).

Candidate HC plasmids for antibody A-G were designed using the complete variable heavy (VH) chain sequence information obtained from the IgG full-length sequencing. In order to design a complete VH chain also for antibodies H and I, for which a complete IgG sequencing was lacking (only the CDR3 sequence was available) and information for the FR1, CDR1, FR2, CDR2, F3 and FR4 were missing, we used the respective sequences derived from antibodies E and F. The antibody LC was specifically designed with a random (not binder) kappa variable light (VL) sequence, to prevent any contribution to antigen binding.

One day before transfection, the cells were seeded at an appropriate density in Corning Erlenmeyer Flasks. On the day of transfection, DNA and transfection reagent were mixed at an optimal ratio and then added into the flask with cells ready for transfection. The recombinant plasmids encoding target protein was transiently transfected into suspension Expi293F cell cultures. The cell culture supernatant collected on day 6 post-transfection was used for purification. Antibodies released in the Expi293F cell supernatant were purified with the AmMag™ Protein A Magnetic Beads kit and resuspended in PBS, pH 7.2, following manufacturer instructions (Genscript, cat. n. L00695). The purified antibodies were analyzed by SDS-PAGE analysis using precast gel (Genscript, Cat.No. M42012) for molecular weight and purity measurements. The concentration was determined by A280 method. Selected candidate antibodies E, G, H and I were also produced in a chimeric murine/human IgG2a/k format, where human-derived VH and VL sequences are in a murine backbone (mIgG2a/k) to avoid non-specific binding to the human Fc-gamma receptor potentially expressed by target cells.

### Cell lines

The nine candidate antibodies (A-I) were tested on different melanoma cell lines and patient-derived xenograft (PDX) cells for their ability to recognize surface antigens (PDX cells were generated at the European Institute of Oncology, IEO, Milan, (29)). Melanoma M14 cells (UCLA‐SO‐M14, RRID: CVCL_1395) were grown in low-glucose DMEM: HAM-F12 1:1 medium (Gibco cat.n.11885084, 11765054) with 10% heat-inactivated Fetal Bovine Serum (FBS, Gibco cat. n.A5256701), 2 mM final concentration of L-Glutamine (Gibco, cat. n. 25030-024), 4ug/mL final concentration of insulin, (Gibco, cat. n.12585014), and a final concentration of 100I.U./mL penicillin and 100μg/mL streptomycin penicillin/streptomycin (p/s, Gibco, cat. n. 15140-122); metastatic melanoma SK-MEL-28 cells (ATCC, HTB-72) were cultured in Eagle’s Minimum Essential Medium (EMEM, ATCC, cat n. 30-2003) with 10% of FBS and p/s, metastatic melanoma PDX cells MM13 (NRASQ61L mutated), MM2 (BRAFV600E mutated) and MM27 (BRAFV600E mutated) (29) were grown in IMDM glutaMAX medium (Gibco, cat. n.31980030) with 10% FBS and p/s for short-term *in vitro* culture (two passages) for antibodies binding tests. Normal Human Epidermal Melanocytes 2 (NHEM 2, Promocell, cat. n.C-12402) were cultured in serum-free, phenol red Melanocyte Growth Medium M2 (Promocell, cat. n.C-24305) supplemented with Growth Medium M2 SupplementMix (Promocell, cat. n. C-39420) following manufacturing culture instructions.

### Antibody binding test and flow cytometry analysis

In a first series of experiments, metastatic melanoma PDX cells and melanoma cell lines were fixed with paraformaldehyde (FA, Cat.J61899.AK,;1% in PBS) for 10 minutes. After blocking with bovine serum albumin (BSA, MERCK, Cat.A9418; 1% in PBS) solution for 30 minutes, candidate antibodies were incubated with target cells at the final concentration of 25 ng/uL in 1% BSA/PBS solution, along with a human IgG1/k isotype antibody (Novus Biologicals, Cat. DDXCH01P-100), used as a control, at the same final concentration. The secondary antibody employed was a goat anti-human IgG Fc-fragment conjugated with the Alexa Fluor 488 fluorophore (Cat. 109-546-008, Jackson Immunoresearch). Best candidates antibodies E, G, H and I, produced in a murine IgG2a/k format, were re-tested on live (non-fixed) cells to confirm their binding properties. In this case, a murine purified Mouse IgG2a, κ Isotype Control (BD Pharmingen, clone G155-178) was used as control, while a goat anti-mouse (H+L) antibody conjugated with Alexa Fluor 488 or Alexa Fluor 647 (Invitrogen, Cat. A32723 and A21235) were used as secondary detection antibodies. Samples were acquired using the Attune NxT Flow Cytometer (Invitrogen, Cat.A24858) connected to an autosampler allowed to process multiple 96-well plates. Flow cytometer data were analyzed by using FlowJo10 software (TreeStar Industries, Ashland, OR).

### Immunohistochemistry analysis of normal and melanoma tissue specimens

The two antibodies (H and I) that were finally selected were tested for binding to the FDA Standard Tissue Array (Cat. T823470, AMSBIO, UK) tissue microarray (TMA), consisting of 87 normal tissue samples, and a melanoma tissue panel containing 41 cases of malignant melanoma and 11 cases of normal skin tissue (Cat. ME481c, AMSBIO, UK). Signal detection was performed by using the Ventana BenchMark ULTRA staining system (Ventana Medical Systems, Tucson, AZ, USA) for fully-automated immunohistochemistry (IHC) workflow. The antibody I was used at a concentration of 20 ng/uL. The staining protocol uses a citrate buffer (pH = 6), blocking with Ventana Antibody Diluent with Casein (Roche) for 8 minutes, and antigenic unmasking at 100 degrees for 24 minutes (ULTRA Cell Conditioning CC2, Roche). The antibody H and control isotype antibody were tested at a concentration of 10 ng/uL. The staining protocol uses EDTA (pH = 8), blocking with Ventana Antibody Diluent with Casein (Roche) for 8 minutes and an antigenic unmasking at 100 degrees for 36 minutes (ULTRA Cell Conditioning CC1, Roche). Binding detection of tested antibodies was performed with ultraView Universal Alkaline Phosphatase Red Detection Kit (Roche). All the tissue sections were counterstained for 16 min with Hematoxylin II (Ventana Medical System) after each immunostaining. The day after the session on the Ventana instrument, slides were removed and placed in a special tray, where they were thoroughly washed with running water and mild soap. This procedure removed the residual liquid coverslip oil (LCS solution) that would otherwise interfere with antibody binding. Next, slides were washed for one minute in demineralized water, and finally dried at 60°C for five minutes on a stove, before being incubated at room temperature for two hours with the antibodies. After drying, slides were soaked for a few seconds in xylene and placed with a drop of Histo Mounting solution (Cat. 008030, Thermo Fisher Scientific, Waltham; MA) before coverslip application and overnight drying at room temperature. High-resolution Whole Slide Imaging (40× magnification) of IHC stained slides were acquired using the Aperio CS2 slide scanner (Leica Biosystems Nussloch GmbH). Staining intensity was evaluated through visual assessment by an expert pathologist (F.L.), and antibody binding was scored as follows:: 0, negative; 0.5, very weak; 1, weak; 2, intermediate; 2.5, intermediate-strong; 3, strong.

### Statistical analysis

All data were analyzed with GraphPad Prism v6.0 (GraphPad Software, La Jolla, CA, United States), and results were represented as mean ± SD. For cytometry analysis data, significance was determined by unpaired t-test (equal variance, two-tailed). For tissue array data, significance was calculated based on Fisher’s exact and Mann-Whitney test. P-values < 0.05 were considered statistically significant. To identify significantly expanded/contracted clonotypes, we use edgeR software package (30). P-value and false discovery rate (FDR) were calculated with exact test in edgeR, as first described by Robinson et al. (31), using trended dispersion estimate. We use the 0.05 threshold on FDR to select clonotypes with significant changes in the concentration.

## Results

### RNAseq analysis of the memory B-cell receptor repertoire identifies candidate anti-melanoma antibodies

**Figure 1:**
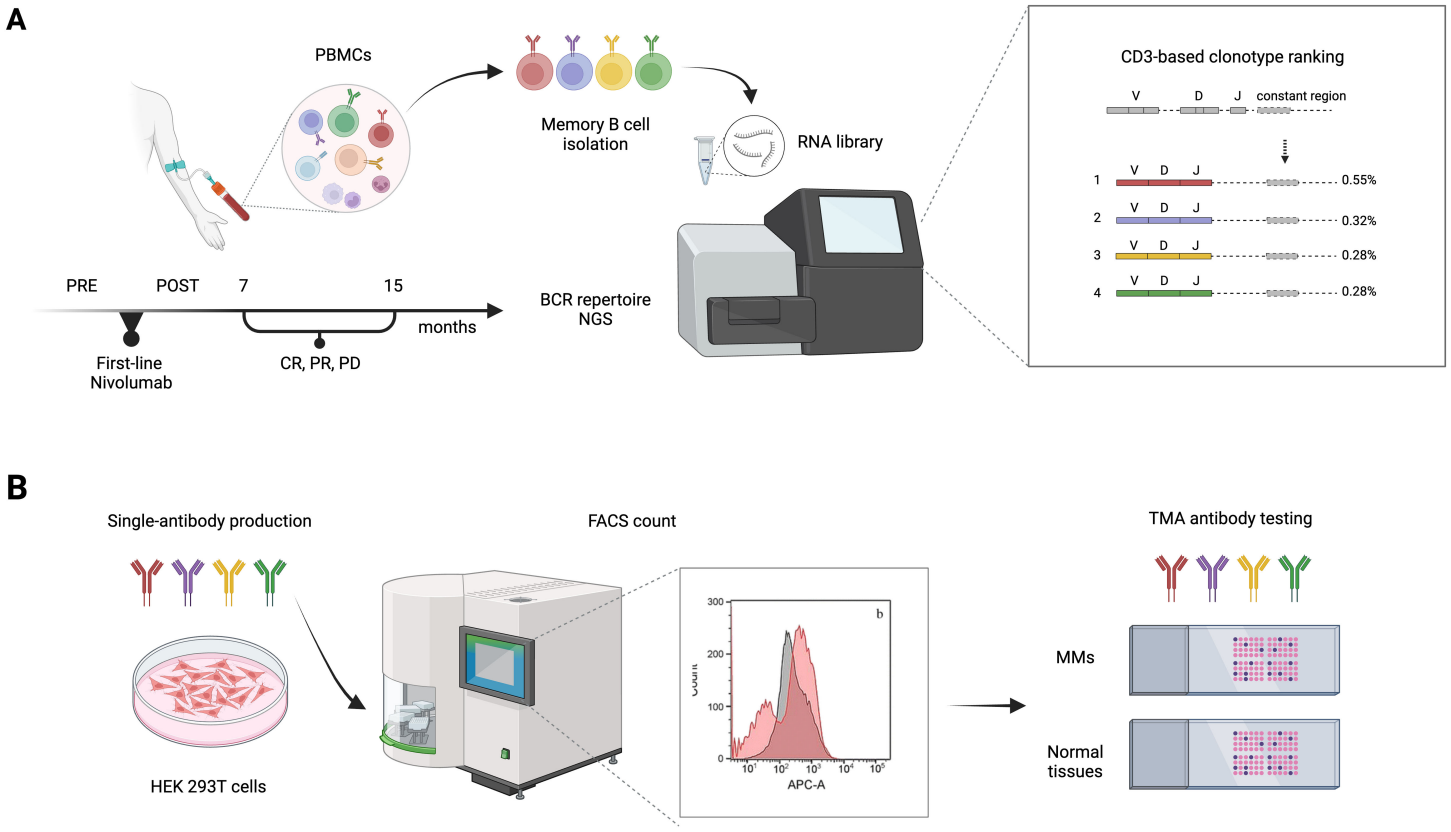
Experimental workflow of BCR repertoire sequence analysis for patient-derived antibody selection and production. **(A)** Memory B cells were isolated from blood samples of non responders (PD), partial responders (PR), or complete responders (CR) metastatic melanoma (MM) patients, before (PRE) and after (POST) first-line therapy with nivolumab. An NGS-based approach was then used to analyze the memory BCR repertoires. V, D, and J (variable–diversity–joining rearrangement) gene usage and their rearrangement, reflecting the variability of the immune responses of different BCR repertoires, was used to define the level of shared antibody sequences between samples and select candidate anti-tumor antibodies. **(B)** Antibody candidates retrieved from the patients’ BCR repertoire sequencing analysis were produced in Expi293F™/ HEK 293T cells and tested for specificity and sensitivity on MM cell lines by FACS and on tumor TMAs. BCR, B-cell receptor; CDR3, complementarity-determining region 3; FACS, fluorescence-activated cell sorting; PBMC, peripheral blood mononuclear cells; NGS, next-generation sequencing; TMA, tissue microarray.

To address the unmet demand for novel immunotherapy approaches that could be coupled with ICI treatments and achieve persistent and effective immune responses, we conceived an innovative screening strategy for the identification of circulating anti-melanoma antibodies from nivolumab-treated patients. The experimental workflow (**Figure 1**) is based on the comparative BCR sequencing analysis of MM patients who responded to the anti-PD-1 versus patients who did not respond to the treatment. In total, six melanoma patients who had either complete response (CR) or partial response (PR) to first-line therapy with nivolumab were included in the B-cell receptor repertoire analysis, as compared with patients who did not respond and experienced progressive disease (PD). Specifically, we processed the PBMCs from MM patients, pre- and post-therapy (seven to fifteen months after therapy start) to isolate memory B cells and extract total RNA for subsequent next-generation sequencing (NGS) analyses (**Table 1**).

We explored all possible polypeptide sequences of the complementarity-determining region 3 (CDR3) of the variable immunoglobulin chain resulting from the V-D-J segment recombination. We focused on specific CDR3 sequences that were enriched, post-treatment versus pre-treatment, looking for immunoglobulins generated *de novo* by B-cell clones that might have arisen in responder patients as a consequence of PD-1 blockage (either partial or complete clinical response). MiXCR software was employed to identify the V, D, J, and CDR3 sequences from the BCR sequencing (32). The analysis and graphical processing of the data generated with MiXCR were conducted with the VDJviz software (28).

A first sequencing run was carried out by prioritizing solely the variable heavy (VH) chain of IgG mRNAs and either focusing on the CDR3 regions (IgG CDR3) or extending the sequencing to the complete VC (full-length IgG). Looking for the IgG CDR3 sequencing, for each sample, we sequenced two separate biological replicates, pre - and post-immunotherapy (**Supplementary Table 1**). Minimal variations of the mean CDR3 nucleotide length have been observed among samples. Also, only a few fractions of sequences were shared for the same patient between the two sequencing replicates and pre- and post-therapy (**Supplementary Figure 1A** **).** Looking for featured CDR3 sequences, exclusively shared in both post-therapy samples, that could distinguish a specific clinical outcome, we analyzed post-therapy replicate samples of PD, PR or CR patients and no common sequences were found (**Supplementary Figure 1B** **).**

**Figure 2:**
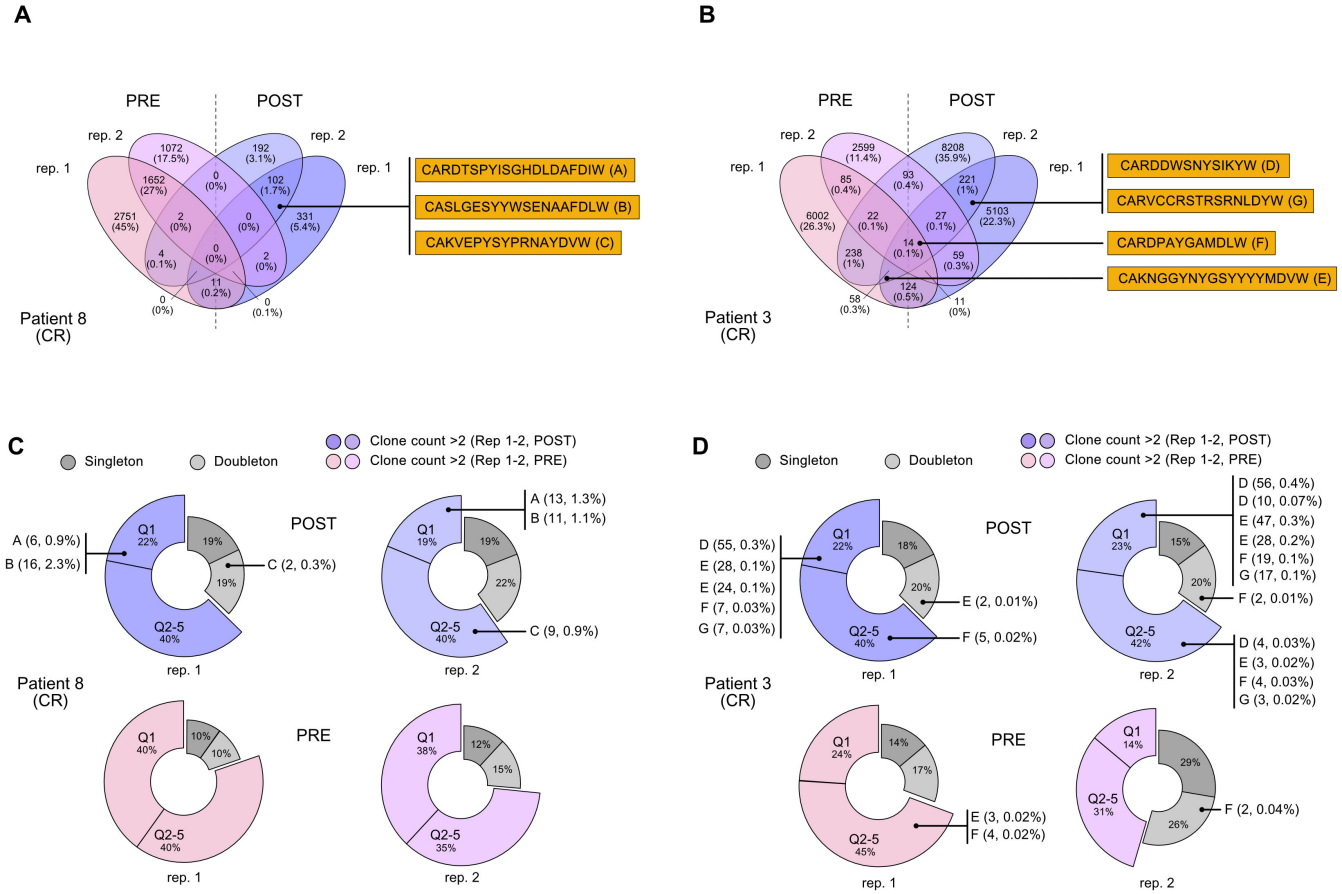
Selection of CDR3 sequences identified through the NGS-based BCR repertoire analysis of IgG sequencing. Selected CDR3 sequences from CR patient n. 8 and CR patient n. 3 were obtained from an IgG-specific sequencing of all samples. **(A, B)** Venn diagrams show common nucleotide sequences that were combined between the two replicate BCR sequencing, pre- (PRE) and post-treatment (POST) with nivolumab. The number of shared sequences is indicated along with the fraction relative to the total counts (in parentheses). Amino acidic sequences of candidate CDR3 for the construction of anti-melanoma antibodies **(A-E)** are highlighted. Also refer to **Table 2**. **(C, D).** Clone count distribution of the two replicate BCR sequencing, pre- (PRE) and post-treatment (POST), for patient n. 8 and patient n. 3. CDR3 sequences that were counted only once (singleton) or twice (doubletone) are depicted in gray. CDR3 that have been detected more than twice (clone count >2) are represented by the 20% quantile distribution, with the most enriched clones being in Q1. Some antibodies can be codified by mismatched nucleotide sequences, and were present at more than one clone count (antibodies D, E, F, G). Clonotype count and relative clonotype frequency is indicated in parentheses. BCR, B-cell receptor; CDR3, complementarity-determining region 3; CR, complete response.

Hence, to identify any possible antibodies that were significantly enriched after immune treatment, including potential *de novo* formed clonotypes, in responder patients, we applied the following criteria for CDR3 sequences selection: being detected only in patients in complete response; being present in both post-therapy replicates; being present in one pre-therapy replicate at most; detection frequency (clone fraction) above 0.1% (**Table 2**). With these principles, we single out seven possible CDR3 sequences for candidate *de novo* anti-melanoma antibodies (A-G), from patient 8 and patient 3 (**Figures 2A, B**). With only three exceptions (sequence C, E, and F), these selected amino acid sequences were derived from mRNA transcripts that were mostly found at a clone count above two (meaning that a distinct CDR3-codifying mRNA sequence has been detected more than twice), in the upper 20% quantile (Q1), with fewer candidate sequences being detected at a lower frequency (Q2-5) as well (**Figures 2C, D**). Interestingly, for patient n.8, the clone abundance distribution exhibited a substantial variation before and after treatment, showing an increase in the total amount of singletons and doubletons (clones that were found at either one or two copies) and a reduction in the number of unique clones detected at higher frequency (Q1), following the immune treatment (**Figure 2C**). By speculation, a decrease in the proportion of CDR3 sequences detected in the top-20% clone count (Q1) might suggest that only a small group of B cell clones is becoming more dominant post-treatment, reflecting an enrichment of few specific sequences. Coherently, a decrease in the clonotype diversity, and an increase of the mean clonotype frequency (**Supplementary Table 1**; **Supplementary Figure 4**) are observed in samples post-therapy. Also, different mRNA sequences encoding for the same antibody were detected at more than one clone count (antibodies D, E, F, and G), suggesting convergence and selection towards the same amino acid sequence from different clonotypes. Detailed information on selected polypeptides from IgG CDR3 sequencing data from patients 3 and 8, including the V, D, and J type of segment used, are reported in **Table 2**.

**Table 2:** Candidate CDR3 sequences for anti-melanoma antibody construction.

Sample	Patient ID	Isotype	Clinical response	Frequency (%)	Count	CDR3 (a.a.)	V	D	J	Antibody ID
8-post-rep1-IGG	8	IgG	CR	0,86	6	CARDTSPYISGHDLDAFDIW	IGHV3-7	IGHD5-12	IGHJ3	A
8-post-rep2-IGG				1,30	13	CARDTSPYISGHDLDAFDIW	IGHV3-7	IGHD5-12	IGHJ3	
8-pre-rep1-IGG				No matches found						
8-pre-rep2-IGG				No matches found						
8-post-rep1-IGG	8	IgG	CR	2,30	16	CASLGESYYWSENAAFDLW	IGHV3-64	IGHD1-26	IGHJ3	B
8-post-rep2-IGG				1,10	11	CASLGESYYWSENAAFDLW	IGHV3-64	IGHD1-26	IGHJ3	
8-pre-rep1-IGG				No matches found						
8-pre-rep2-IGG				No matches found						
8-post-rep1-IGG	8	IgG	CR	0,29	2	CAKVEPYSYPRNAYDVW	IGHV4-34	IGHD3-10	IGHJ3	C
8-post-rep2-IGG				0,88	9	CAKVEPYSYPRNAYDVW	IGHV4-34	IGHD3-10	IGHJ3	
8-pre-rep1-IGG				No matches found						
8-pre-rep2-IGG				No matches found						
3-post-rep1-IGG	3	IgG	CR	0,26	55	CARDDWSNYSIKYW	IGHV4-39	IGHD3-10	IGHJ4	D
3-post-rep2-IGG				0,39	56	CARDDWSNYSIKYW	IGHV4-39	IGHD3-10	IGHJ4	
				0,07	10	CARDDWSHYSIKYW	IGHV4-39	IGHD3-10	IGHJ4	
				0,03	4	CARDDWSNYSIKYW	IGHV4-39	IGHD3-10	IGHJ4	
3-pre-rep1-IGG				No matches found						
3-pre-rep2-IGG				No matches found						
3-post-rep1-IGG	3	IgG	CR	0,13	28	CAKNGGYNYGSYYYYMDVW	IGHV1-69	IGHD5-18	IGHJ6	E
				0,11	24	CAKNGGYNYGSYYYYMDVW	IGHV1-69	IGHD5-18	IGHJ6	
				0,01	2	CAKNGGYNYGSYYYYMDVW	IGHV1-69	IGHD5-18	IGHJ6	
3-post-rep2-IGG				0,32	47	CAKNGGYNYGSYYYYMDVW	IGHV1-69	IGHD5-18	IGHJ6	
				0,19	28	CAKNGGYNYGSYYYYMDVW	IGHV1-69	IGHD5-18	IGHJ6	
				0,02	3	CAKNGGYNYGSYYYYMDVW	IGHV1-69	IGHD5-24	IGHJ6	
3-pre-rep1-IGG				0,02	3	CAKNGGYNYGSYYYYMDVW	IGHV1-69	IGHD5-18	IGHJ6	
3-pre-rep2-IGG				No matches found						
3-post-rep1-IGG	3	IgG	CR	0,03	7	CARDPAYGAMDLW	IGHV3-7	IGHD5-24	IGHJ3	F
				0,02	5	CARDPAYGAMDLW	IGHV3-7	IGHD4-17	IGHJ3	
3-post-rep2-IGG				0,13	19	CARDPAYGAMDLW	IGHV3-7	IGHD4-17	IGHJ3	
				0,03	4	CARDPAYGAMDLW	IGHV3-7	IGHD5-24	IGHJ3	
				0,01	2	CARDPAYGAMDLW	IGHV3-7	IGHD4-17	IGHJ3	
3-pre-rep1-IGG				0,02	4	CARDPAYGAMDLW	IGHV3-7	IGHD4-17	IGHJ3	
3-pre-rep2-IGG				0,04	2	CARDPAYGAMDLW	IGHV3-7	IGHD5-24	IGHJ3	
3-post-rep1-IGG	3	IgG	CR	0,03	7	CARVCCRSTRSRNLDYW	IGHV1-2	IGHD2-21	IGHJ4	G
3-post-rep2-IGG				0,12	17	CARVCCRSTRSRNLDYW	IGHV1-2	IGHD2-21	IGHJ4	
				0,02	3	CARVCCRSTRSRNLDYW	IGHV1-2	IGHD2-2	IGHJ4	
3-pre-rep1-IGG				No matches found						
3-pre-rep2-IGG				No matches found						
7-post-rep1-IGH	7	IgM	PR	4,7	202	CARDASSGSYAGRAHFDYW	IGHV1-2	IGHD1-26	IGHJ4	H
7-post-rep2-IGH				6,5	202	CARDASSGSYAGRAHFDYW	IGHV1-2	IGHD1-26	IGHJ4	
7-pre-rep1-IGH				No matches found						
7-pre-rep2-IGH				No matches found						
8-post-rep1-IGH	8	IgM	CR	0,32	15	CARHRRAGAHFFDYW	IGHV4-39	IGHD6-19	IGHJ4	I
8-post-rep2-IGH				0,28	17	CARHRRAGAHFFDYW	IGHV4-39	IGHD6-19	IGHJ4	
8-pre-rep1-IGH				No matches found						
8-pre-rep2-IGH				No matches found						

Amino acidic (a.a.) sequence information on selected polypeptides from the IgG CDR3 sequencing analysis of patients 3 and 8, are reported, along with the V, D, and J segment type. CDR3 sequences selection for the antibodies (A to I) production was based on the presence only in those patients with complete response (CR) and partial response (PR), and with a detection frequency (as referred to the clone count over the total number of reads) above 0.1%.

**Figure 3:**
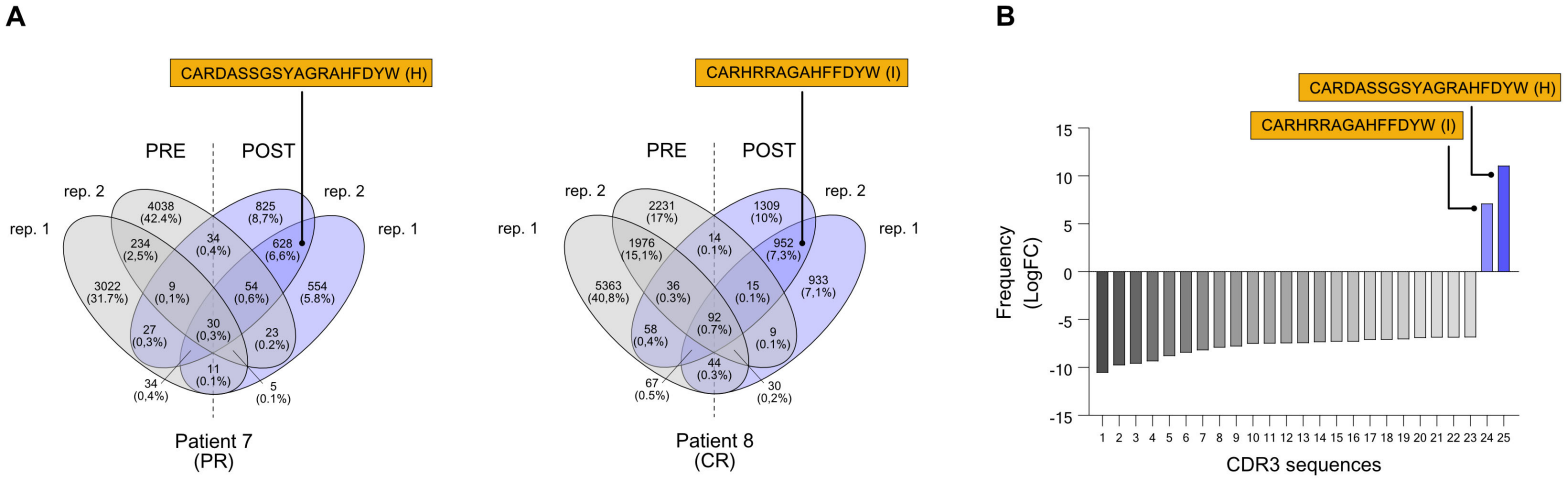
Selection of CDR3 sequences identified through the NGS-based BCR repertoire analysis of IgH sequencing. Selected CDR3 sequences from CR patient n. 7 and CR patient n. 8 were obtained from an isotype-independent complete heavy chain immunoglobulin sequencing. Also refer to **Table 2**. **(A)** Venn diagrams show common nucleotide sequences that were combined between the two replicate BCR sequencing, pre- (PRE) and post-treatment (POST) with nivolumab. The number of shared sequences is indicated along with the fraction relative to the total counts (in parentheses). Amino acidic sequences of candidate CDR3 for the construction of anti-melanoma antibodies (H and I) are highlighted. **(B)** Frequency distribution, indicated as logarithmic fold change (LogFC), of the 25 significantly different CDR3 sequences. Only two sequences (H and I) were positively enriched post-treatment, while absent pre-treatment; the other 23 CDR3 sequences that were found pre-treatment were absent post-treatment (negative LogFC). Also refer to **Table 3**. BCR, B-cell receptor; CDR3, complementarity-determining region 3; PR, partial response; CR, complete response.

During the second sequencing run, all heavy chain CDR3 sequences (IgH CDR3) were sequenced without any pre-selection, irrespective of their isotype (**Supplementary Table 2**). From IgH CDR3 sequencing, we observed that IgM represented the most frequent Ig isotype (47,2-79,5%) in the memory B cell compartment we have isolated, followed by IgA (7,3-30,2%), IgD (5,1-11,5%), while IgG was less abundant (3,8-11,2%), and IgE was not detected at all; allegedly, there was no evident difference in terms of Ig isotype frequency either between responders and non-responders, or pre- and post-therapy (**Supplementary Figure 2**). Similar to the IgG CDR3 sequencing, also the IgH CDR3 exhibited minimal overlap (less than 1%) in CDR3 sequences between the two replicates (pre- and post-treatment) for all samples and no common sequences between post-treatment replicates, suggesting the absence of a distinct CDR3 signature that could be linked to the clinical response (**Supplementary Figure 3**). Therefore, similar to our approach for the IgG CDR3 sequencing and applying the same selection criteria (this time including also sequences from PR patients), we examined the IgH CDR3 repertoire in pre- and post-treatment sequencing replicates, looking for enriched/*de novo* formed clonotypes after ICI treatment. We found two CDR3 sequences (H and I) that were exclusive to patient 7 (PR) and patient 8 (CR) after treatment (**Figure 3A**). By employing rigorous statistical analysis, a total of 25 sequences showed a drastic variation between pre and post ICI-treatment. In particular, 23 sequences were not detectable (0 read counts) post treatment while 2 sequences (called H and I) were clearly *de novo* formed (see **Table 3**). Interestingly, H and I sequence were the only ones that reached a statistical significance out of the 628 and 952 sequences from patients 7 and 8 shared sequences, respectively (**Figure 3B**), demonstrating specific enrichment after nivolumab treatment. Both candidate sequences H and I, identified as IgM isotypes, were reengineered into IgG/k antibodies, in line with candidate CDR3 A-G.

**Table 3:** Memory BCR repertoire with IgH sequencing identifies CDR3 sequences enriched in responder MM patients.

Patient ID	Isotype	rep1 PRE	rep2 PRE	rep1 POST	rep2 POST	logFC	P-Value	FDR	CDR3 (aa)
**7**	**IgM**	**0**	**0**	**202**	**202**	**11,07**	**5,10E-06**	**3,60E-02**	**CARDASSGSYAGRAHFDYW**
**8**	**IgM**	**0**	**0**	**15**	**17**	**7,1**	**1,90E-05**	**1,61E-02**	**CARHRRAGAHFFDYW**
8	IgM	22	9	0	0	-6,85	7,12E-05	4,59E-02	CVKSPYSSNSMW
8	IgM	20	11	0	0	-6,86	7,00E-05	4,59E-02	CVKSPYSSGQHW
8	IgA	19	12	0	0	-6,87	6,94E-05	4,59E-02	WAREDLYGSESRYIAYW
8	IgM	23	9	0	0	-6,9	5,47E-05	4,11E-02	CARHPTGFPNWFDPW
8	IgM	22	13	0	0	-7,04	2,43E-05	1,93E-02	CVKSPYSSSSMW
8	IgM	29	8	0	0	-7,09	1,84E-05	1,61E-02	CARSVVFTGGFDYW
8	IgA	22	15	0	0	-7,12	1,44E-05	1,39E-02	WARDSTGPPSGGRYW
8	IgM	23	18	0	0	-7,28	5,36E-06	5,58E-03	CARDMDTYNRFDYW
9	IgA	23	18	0	0	-7,29	1,83E-10	7,33E-07	WARDFTSMRGVDVFDVW
8	IgM	33	11	0	0	-7,35	4,10E-06	4,63E-03	CARDLDYYYGMDVW
8	IgM	28	18	0	0	-7,43	2,14E-06	2,64E-03	CARHPIARHPSDYW
8	IgM	38	10	0	0	-7,46	1,74E-06	2,36E-03	CARVPSENNGYAPDYW
8	IgG	25	22	0	0	-7,48	1,69E-06	2,36E-03	CSSVYDTSSSYYLEHW
8	IgA	23	25	0	0	-7,52	1,14E-06	1,93E-03	CVGRTNGQYW
9	IgA	33	25	0	0	-7,79	1,83E-15	1,10E-11	CARGPNLWTEPVHGMDVW
8	IgG	35	29	0	0	-7,92	6,60E-08	1,28E-04	CAGDSRGYLAYW
8	IgA	241	168	1	0	-8,18	1,94E-20	1,31E-16	CARDSTGPPSGGRYW
8	IgA	61	33	0	0	-8,45	6,21E-10	1,40E-06	WARERTTFGSVRGGFDVW
9	IgA	52	64	0	0	-8,79	1,90E-28	2,29E-24	CARDFTSMRGVDVFDVW
8	IgA	88	82	0	0	-9,33	3,06E-14	8,28E-11	CAREDLYGSESRYIAYW
8	IgD	152	56	0	0	-9,58	1,43E-15	4,85E-12	CARVPSEYNGYAPDYW
8	IgA	118	112	0	0	-9,77	1,34E-16	6,05E-13	CARERTTFGSVRGGFDVW
8	IgG	239	156	0	0	-10,53	2,64E-21	3,58E-17	CAPRRFGGYEFL

CDR3 sequences, depicted by their amino acid sequence, were ranked according to the logarithm fold change (LogFC) between pre-treatment (PRE) and post-treatment (POST) in both replicas. Antibodies derived from CDR3 sequences H (patient 7) and I (patient 8), significantly enriched after nivolumab therapy (highlighted in bold), were designed and produced to test their ability to target melanoma cells. FDR, false discovery rate.

Finally, it is worth noting that a substantial rise in the average clonotype frequency was evident following the administration of nivolumab across all the samples, regardless of the clinical outcome (**Supplementary Figure 4**). These findings suggest that immunotherapy could potentially boost the production of B cells, enabling clonal selection of anti-melanoma antibodies.

### Responder patients-derived candidate antibodies can target melanoma cells

**Figure 4:**
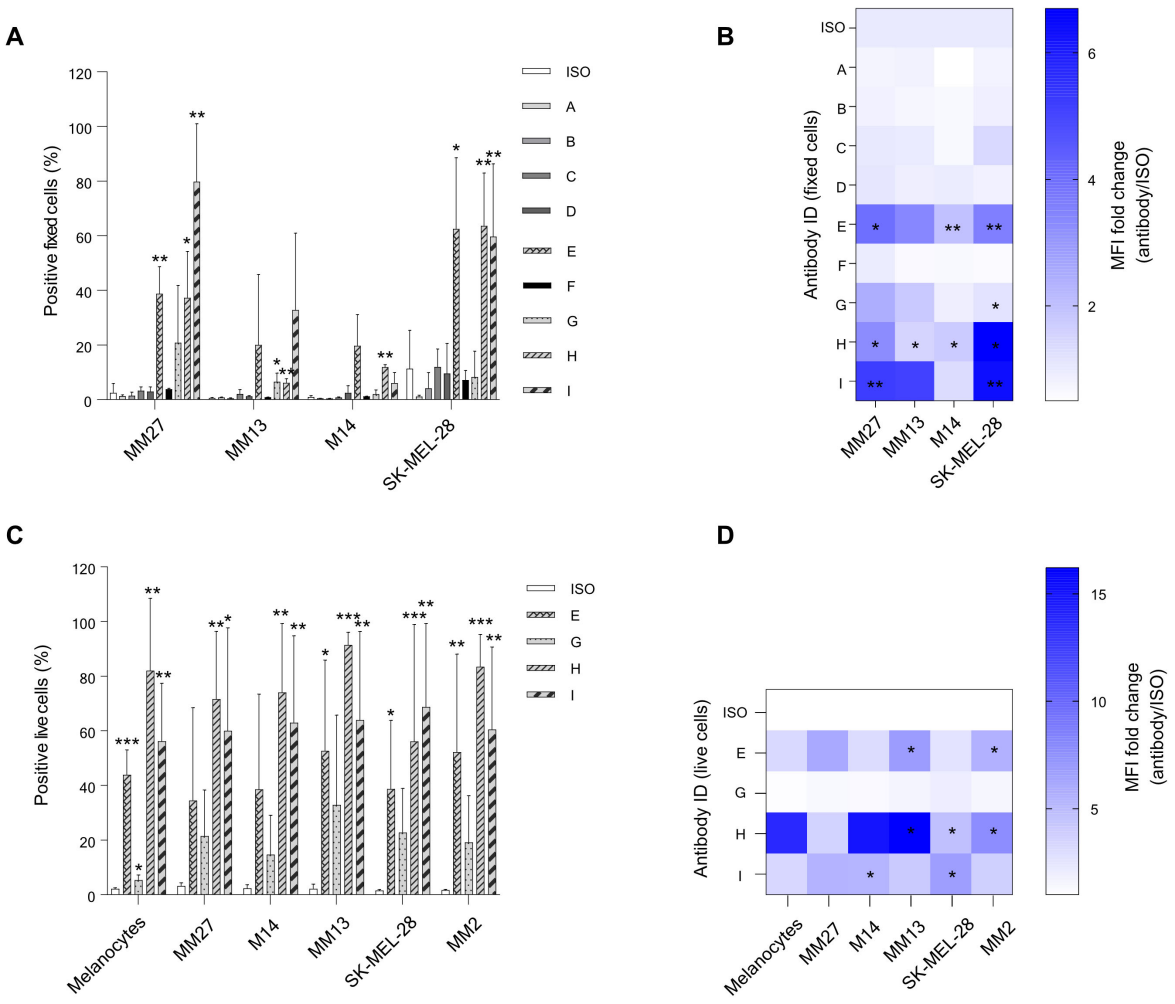
Flow cytometry evaluation of the binding capacity for selected candidate antibodies on different melanoma cells. **(A, B)** First candidate antibodies A-I (human IgG1/k) were tested on paraformaldehyde-fixed metastatic melanoma (MM) patient-derived xenograft (PDX) cell lines (MM13 and MM27) and MM commercial cell lines (SK-MEL-28 and M14). **(C, D)** Selected candidates E, G, H, and I (chimeric human/murine IgG2a/k) from the preliminary test **(A-B)**, were further evaluated for their binding affinity on live PDX cells (MM2, MM13 and MM27), commercial MM cells (M14 and SK-MEL-28), and normal melanocytes. Antibody binding affinity was calculated both as a percentage of positive cells and as median fluorescence intensity (MFI), represented as fold changes (FC) relative to the isotype antibody (ISO) control (mean values and standard deviation of at least 2 independent experiments). *P ≤ 0.05, **P ≤ 0.01, ***P ≤ 0.001.

**Figure 5:**
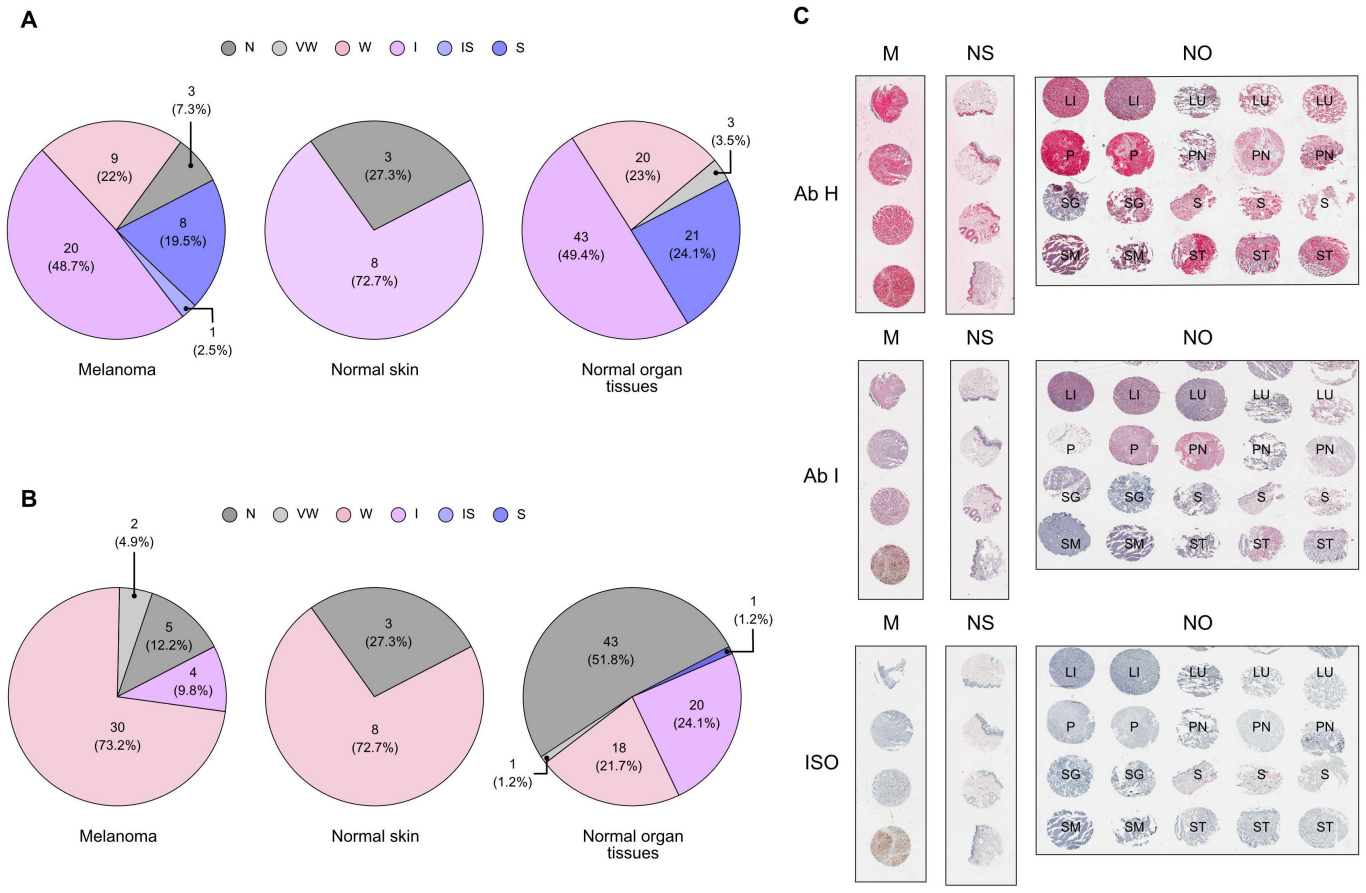
Validation of candidate antibody binding affinity on melanoma tissues. Immunohistochemical analysis of antibody H **(A)** and antibody I **(B)** on a panel of melanoma (M), normal organ (NO), and normal skin (NS) tissue microarray (TMA), in comparison with the isotype antibody (ISO) control staining. Representative examples of TMA areas of the sections stained are included **(C)**. Each sample was classified according to a staining score graded as follows: 0, negative (N); 0.5, very weak (VW); 1, weak (W); 2, intermediate (I); 2.5, intermediate-strong (IS); 3, strong (S). Pie charts represent count and fraction of the grade of antibody staining. Every TMA picture was acquired at the same magnification (Scale bar = 200 μm). (LI, liver; LU, lung; P, pancreas; PN, peripheral nerve; SG, salivary gland; S, skin; SM, skeletal muscle; ST, stomach). Related to **Supplementary Figure 5** and **Supplementary Table 3**.

After choosing candidate CDR3 sequences from the BCR repertoire analysis, we generated a set of 9 fully human hIgG1/k antibodies (A to I) in Expi293F producer cells with a random (not binder) kappa variable light chain (the same one for all candidates) and antibody-specific VH chain for each candidate antibody. These patient-derived antibodies were examined for their ability to bind to melanoma cell lines and PDX cells (**Figures 4A, B**). We selected an assortment of cellular models, including cell lines and PDX, that exhibit a variety of phenotypes, in order to closely mimic the molecular diversity of our tumor cases. In fact, the patients chosen for this study presented with divergent histopathological features, and those individuals with identical diagnoses exhibited different responses to therapy (**Table 1**). Specifically, PDXs are considered a most reliable translational model for assessing patient clinical behavior. Antibody binding to target cells was assessed by fluorescence-activated cell sorting (FACS) cytometry. Candidate antibodies E, G, H, and I were selected for further investigation based on their significant divergence from the isotype control in terms of both the percentage of positively-stained cells and the median fluorescence intensity (MFI) of the antibody signal (**Figures 4A, B**), to be also produced in a chimeric murine/human IgG2a/k format. Thus, we conducted another assessment of the binding affinity of chimeric antibodies E, G, H, and I on melanoma cell lines and PDX cells, with the addition of normal melanocytes. Although able to bind to normal melanocytes too, antibodies E, H, and I confirmed a significant binding affinity for melanoma and PDX cells over the isotype control, both in terms of percentage positivity and MFI (**Figures 4C, D**). Antibodies H and I were specifically assessed by IHC on human tissue microarrays (TMA) of normal tissues (FDA Standard Tissue Array), containing various organs (N = 87), normal skin (N = 11), and melanoma samples from stage I to stage IV (N = 41). Antibody staining signal strength was classified on a scale from negative to strong (**Supplementary Figure 5**; **Supplementary Table 3**). We employed Fisher’s exact and χ2 tests to evaluate the correlation between the histological affinity of the two antibodies in malignant melanoma tissues compared to normal tissues and normal skin (**Supplementary Table 4**). The results revealed that antibody H had a higher binding affinity to the melanoma TMA compared to antibody I: 93% of samples exhibited weak to strong staining, with and 7% displaying very weak and negative staining. When tested on the normal skin panel, antibody H displayed a clear binding, with approximately 73% of samples showing intermediate staining. On normal organ tissues, antibody H was detected at weak to strong level in almost all samples (**Figure 5B**). The comparison of antibody H levels between melanoma TMA and normal organ tissues, as well as normal skin TMA, did not yield any significant differences (**Supplementary Table 4**). Antibody I had a negative-to-very-weak binding in 53% of normal tissues, with 21.7% of samples showing weak staining, 24% displaying intermediate staining, and only 1.2% exhibiting intermediate to strong staining. Conversely, in the melanoma TMA, the substantial majority melanoma cases (>82%) showed positive staining, ranging from weak to strong intensity, with less than 18% of samples showing negative or very weak intensity (**Figure 5B**), obtaining a significant statistical difference when compared to the normal-tissue panel (P-value: 0.0003). Although there was no notable staining in the normal skin tissues (more than a quarter of samples lacked positive staining and about 73% displayed weak staining), the difference with the melanoma TMA was not significant (**Figure 5B**; **Supplementary Table 4**). Finally, there was no correlation between increased antibody staining and melanoma staging (Mann-Whitney test of stage I to IIB compared to stage III-IV, P value > 0,05, data not shown).

## Discussion

We conducted a comprehensive BCR repertoire sequence analysis of memory B cells isolated from the blood of patients with metastatic melanoma. By comparing the repertoire (CDR3 sequencing) of non-responders, partial responders, and complete responders, before and after first-line therapy with nivolumab, we revealed the presence and a means of selection for MM targeting antibodies associated with the treatment and positive clinical outcome. Interestingly, we observed an overall increase in the average clonotype frequency and a decrease of clonotype diversity following the administration of nivolumab in all patients, independently of their clinical outcome.

Several studies support a positive role for B cells within TLSs in several solid tumors (16, 17, 33) including melanoma (11, 34, 35). In the tumors of patients experiencing a clinical response compared to those who do not, B cell marker genes show the most significant differences in expression levels, indicating that switched memory B cells are enriched in the tumors of responders (17). The fact that responders’ tumors typically reveal a significantly greater abundance of switched memory B cells *in situ*, suggests that a trace of this adaptive immune response could be intercepted also in the peripheral blood of those patients. This enrichment of B cells clonotypes in melanoma patients’ tumors also associates with a strong clonal and T cell infiltrate in responders to nivolumab monotherapy (36). Melanoma is a cancer that responds very well to ICI (3, 37, 38), and our findings suggest that nivolumab may indirectly boost the production of B cells and enable clonal selection of antibodies able to opsonize melanoma or to function as boosters for dendritic cells uptake of tumor antigens. The enhancement of B cell function in patients with cancer might expose antibodies that are able to recognize specifically cancer cells (39), and the ability to identify these cancer antigen-reactive antibodies may be of critical clinical importance for limiting the likelihood of tumor growth. Strategically, we decided to focus our sequencing analysis at first on VH CDR3s in order to have a deeper and more comprehensive representation of clonotypes’ frequency and the information on the specific isotype. Afterwards, we performed a full length IgG sequencing (lower depth) that was used to match the ranked CDR3 clonotypes we had selected as putative candidates. This combination allows on one hand to increase the likelihood of selecting true enriched and specific antibodies and to retrieve the correct VH full length sequence for antibody reconstruction. This pipeline allowed the generation of 9 human derived recombinant antibodies. A recent work shows that memory B cells are particularly enriched in tumor tissues compared to the blood of melanoma patients (40). Authors reveal that these tumor-associated B cells undergo clonal expansion and class switch, and, compared with B cells retrieved from patients’ blood, they also produce antibodies that are polyreactive and characterized by autoantigen recognition. Our patient-derived anti-melanoma candidate antibodies, when evaluated by flow cytometry, beside their capacity of binding different melanoma cells with high affinity, also showed a significant binding to normal melanocytes and also other normal tissues. B cells are not passive participants in the immune response, being directly involved in the tumor progression and treatment outcome (11, 35). In this regard, class switching might play a critical role in the production of circulating antibodies that are tumor-specific but also self-reactive, influencing the clinical course (41). A number of studies provide support for the notion that melanoma is associated with the expression of antibodies that have polyreactive traits (37). In fact, serum antibodies can exhibit reactivity towards antigens related to autoimmune diseases, beside cancers, showing an increased amount in patients with ongoing disease compared to those in a post-operative state (40). Systemic immune reactions to self-antigens during immunotherapy in melanoma patients are commonly reported (42, 43). For example, vitiligo develops quite frequently in patients who are being treated with ICIs (44, 45). It is possible that the antibodies derived from our patients could have originated from B cells that underwent a steep clonal expansion, and to some extent, resume aspects of those autoimmune responses. However, the balance between toxicity and clinical benefit most likely depends not only on the cross-reactivity, but also on the concentration and distribution of the antibodies involved. Toxicities and immune related adverse events (irAE) in melanoma patients treated with ICIs are common and often managed by corticosteroids, and their mechanistic origin is not well characterized. T and B cell responses could contribute to the insurgence of ICI toxicities. However, some irAEs can be permanent at the level of the skin and the endocrine system, therefore a careful assessment of the potential tissue cross-reactivity of antibodies is crucial to restrain potentially harmful occurrences once *in vivo* (46). To further validate our responder-derived candidate antibodies for targeting melanoma and normal tissues, we selected antibody H and I for their highest reactivity for melanoma cell lines and whose sequences were the only two uprising with high significance after treatment, suggesting they might have produced *de novo* in response to nivolumab stimulation. These antibodies, besides being very reactive against different melanoma samples, also displayed a certain affinity for normal skin and various health tissues.

The screening of circulating memory B cells in melanoma patients to identify the presence of immunoglobulins targeting tumors has long been pursued. Most relevant studies have revealed that the occurrence of melanoma-specific antibodies is significantly higher in melanoma patients than in healthy volunteers, and these antibodies are enriched in the peripheral blood of cancer patients (47). The antibody response to melanoma, however, diminishes with disease progression, along with a reduction of memory B cells in circulation (40, 48). A hypothesis could be that reduction of circulating memory B cells may be a consequence of the increased production of activated plasma cells or migration of memory B cells from peripheral blood into the tumor environment (40).

We haven’t investigated the tumor tissue, but we believe that a novelty of our study stems from having compared the circulating BCR repertoire of melanoma patients treated with immunotherapy in a neoadjuvant clinical setting, which should maximize the chances of retrieval for these biologics. Our goal was to leverage on the patient’s induced immune response by first-line nivolumab to identify novel therapeutic antibodies against melanoma. By specifically sequencing the CDR3 repertoire of mature memory B cells in patient blood pre- and post-immunotherapy we were expecting to find some immunoglobulin clones that were arising *de novo*, despite a global reduction of circulating B cells in patient compared to healthy donors is expected (40, 47, 48). Although we haven’t measured the effective presence of the antibodies in the responders’ blood or assessed accumulation of antibodies on tumor cells or in TLS *in situ*, the data support the possibility that tumor opsonization might occur favoring the engagement of innate immune cells (i.e. NK cells) or complement dependent cytotoxicity (CDC) thereby contributing to a positive clinical response to nivolumab.

We acknowledge some limitations of our study: the restricted number of patients examined; the absence of matched tumor samples to assess the BCR repertoire *in situ*; relying on the VH sequence for target recognition, only; the reconstruction of some antibodies in a murine/human hybrid format that could have impaired the original binding function of the fully human counterparts, and the unknown nature of the antigens bound. Despite those limits, our results suggest that it is possible to retrieve antibodies endowed with a wide cross-reactivity among several patients, meaning that some inherent features are conserved. Validating these results in a larger patient cohort would be valuable and would further support our hypothesis that it is possible to identify anti-tumor antibodies in responsive patients.

In addition, the intrinsically unbiased and agnostic screening strategy we envisioned could allow the selection of antibodies against unknown and unpredictable targets based on current knowledge but recognized as an important target in a complex context like a patient under treatment. We believe those aspects temper the limitations on the number of patients examined and actually empowers the concept that deeper sequencing analysis in restricted cohorts of patients could be sufficient to find many targeting antibodies with potential application for many patients. As shown in previous studies, we observe a positive binding to both melanoma cells and melanocytes (40). This on-target off-tissue binding could correlate with potential vitiligo, which is commonly considered a good proxy for immunotherapy efficacy. The identification of the target antigens is imperative to grant clinical application to our candidate antibodies and will be the subject of further investigations.

In conclusion, by analyzing the CDR3 output of circulating memory B cells derived from patients with metastatic melanoma, we present findings indicating a specific enrichment of IgG and IgM type antibodies, present in patients’ blood exclusively after nivolumab therapy. Nivolumab might have enhanced a preexisting autoimmune response, underestimated in our analysis, as well as promoted the development of *de novo* clonotypes both endowed with the ability to opsonize tumors to recruit innate immune cells and functions against tumor cells. Our antigen-unbiased screening strategy could potentially be used to further explore the circulating B-cell-associated humoral response to MM or other solid tumors, with the aim of identifying fully human antibodies that may be associated with a positive clinical outcome. These findings provide a basis for future studies to assess the therapeutic effectiveness of the identified antibodies in innovative combinatorial immunotherapy approaches with ICIs. While antibody candidates H and I demonstrated promising tumor binding, their cross-reactivity with normal tissues - particularly melanocytes and skin - raises important concerns regarding specificity and safety. Further characterization of these antibodies is therefore essential to better define their therapeutic index and to minimize potential off-target effects. In this context, future studies should include comprehensive specificity profiling using a broader panel of normal tissues. Additionally, functional assays such as antibody-dependent cellular cytotoxicity (ADCC), complement activation, and *in vivo* efficacy and toxicity models will be crucial to fully assess both the therapeutic potential and safety of these candidates. Such analyses will help determine whether the observed antitumor reactivity can be harnessed for clinical application while maintaining an acceptable safety profile.

## Data Availability

The original contributions presented in the study are publicly available. This data can be found here: https://doi.org/10.6084/m9.figshare.30305437.v1.

## Supplementary material

Supplementary Figure 1 Comparison of CDR3 sequences between melanoma patients following the IgG CDR3 sequencing. **(A)** Venn diagrams showing CDR3 sequences overlap between the two replicates pre- (PRE) and post-treatment (POST) with nivolumab, from IgG CDR3 sequencing. **(B)** CDR3 sequences were matched based on the clinical response for each individual patient, considering only those sequences found post-treatment and overlapping the two sequencing replicates. Percentages in parentheses represent the fraction of CDR3 counts relative to the total count for each patient. BCR, B-cell receptor; CDR3, complementarity-determining region 3; PD, progressive disease; PR, partial response; CR, complete response.

Supplementary Figure 2 Memory B cell immunoglobulin isotype analysis. Immunoglobulin isotype distribution (IgG, IgM, IgD, IgA and IgE) in the memory B cell fraction obtained from IgH CDR3 sequencing of melanoma patients, pre- and post-immunotherapy, represented as the average percentage of the two replicates. PD, progressive disease; PR, partial response; CR, complete response.

Supplementary Figure 3 Comparison of CDR3 sequences between melanoma patients following the IgH CDR3 sequencing. **(A)** Venn diagrams showing CDR3 sequences overlap between the two replicates pre- (PRE) and post-treatment (POST) with nivolumab, from IgH CDR3 sequencing. **(B)** CDR3 sequences were matched based on the clinical response for each individual patient, considering only those sequences found post-treatment and overlapping the two sequencing replicates. Percentages in parentheses represent the fraction of CDR3 counts relative to the total count for each patient. BCR, B-cell receptor; CDR3, complementarity-determining region 3; PD, progressive disease; PR, partial response; CR, complete response.

Supplementary Figure 4 Diversity, mean frequency, and Q1 enrichment from CDR3 IgG and IgH sequencing. Total clonotype diversity, mean clonotype frequency, and number of clonotypes in the upper 20% quantile (Q1) were represented as the average +SD of the two replicates for the IgG CDR3 **(A)** and IgH CDR3 **(B)** sequencing, comparing pre- (PRE) and post-treatment (POST) with nivolumab. PD, progressive disease; PR, partial response; CR, complete response.

Supplementary Figure 5 Antibody testing on tissue microarray. Complete panel of the tissue microarray (TMA) of normal organs (NO), melanoma (M) and normal skin (NS) samples, stained with either antibody H or I, and isotype control antibody. Related to **Supplementary Table S3**.
