# Supplementary figures and images for: Circulating memory B-cell receptor repertoire analysis identifies novel candidate antibodies against metastatic melanoma in immunotherapy-responsive patients
Source: Front Immunol. 2025 Oct 9;16:1636722. doi: 10.3389/fimmu.2025.1636722 (PMC12548061; doi:10.3389/fimmu.2025.1636722)

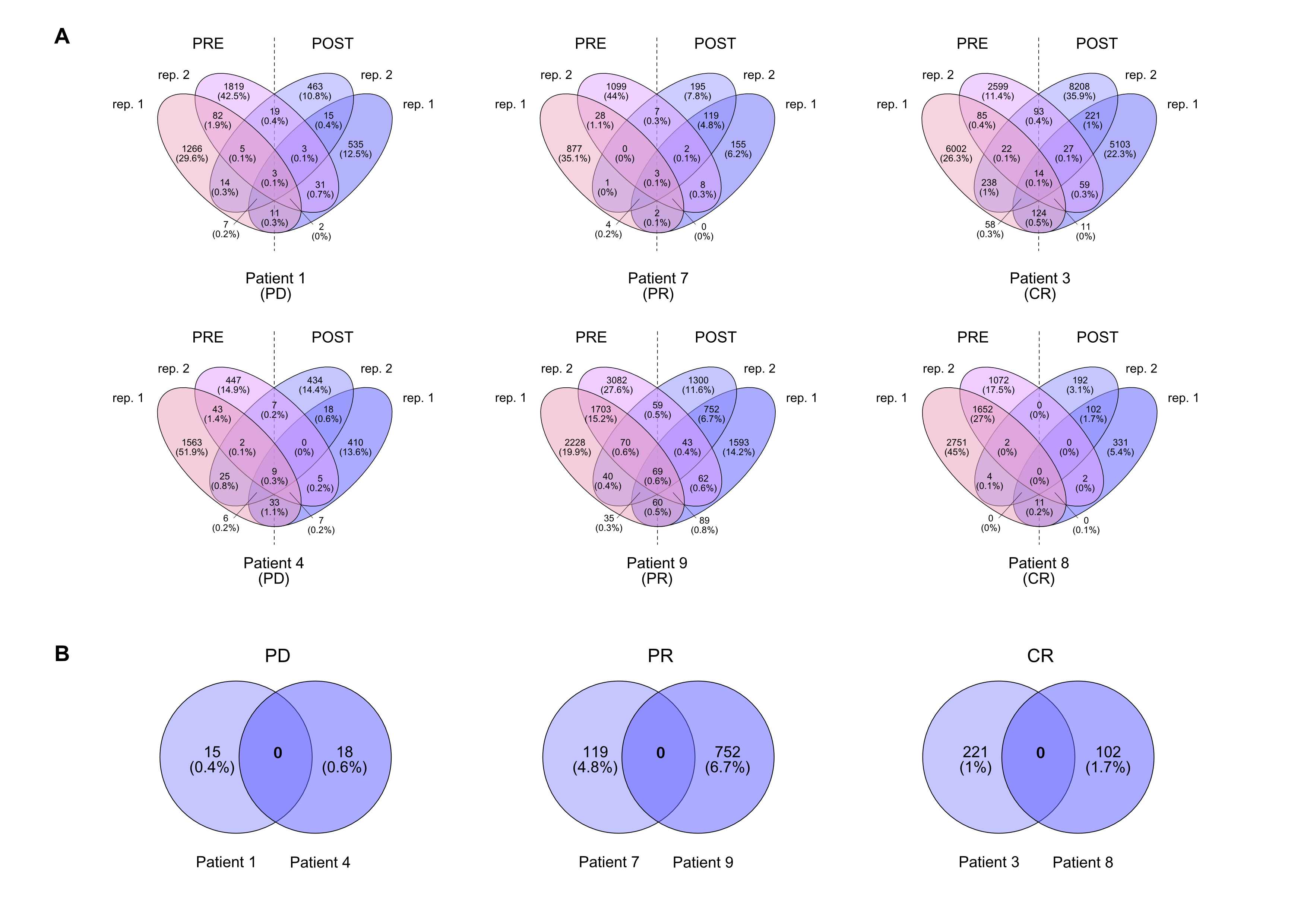

Supplement: Supplementary Figure 1 — Comparison of CDR3 sequences between melanoma patients following the IgG CDR3 sequencing. (A) Venn diagrams showing CDR3 sequences overlap between the two replicates pre- (PRE) and post-treatment (POST) with nivolumab, from IgG CDR3 sequencing. (B) CDR3 sequences were matched based on the clinical response for each individual patient, considering only those sequences found post-treatment and overlapping the two sequencing replicates. Percentages in parentheses represent the fraction of CDR3 counts relative to the total count for each patient. BCR, B-cell receptor; CDR3, complementarity-determining region 3; PD, progressive disease; PR, partial response; CR, complete response. [file Image1.jpeg]

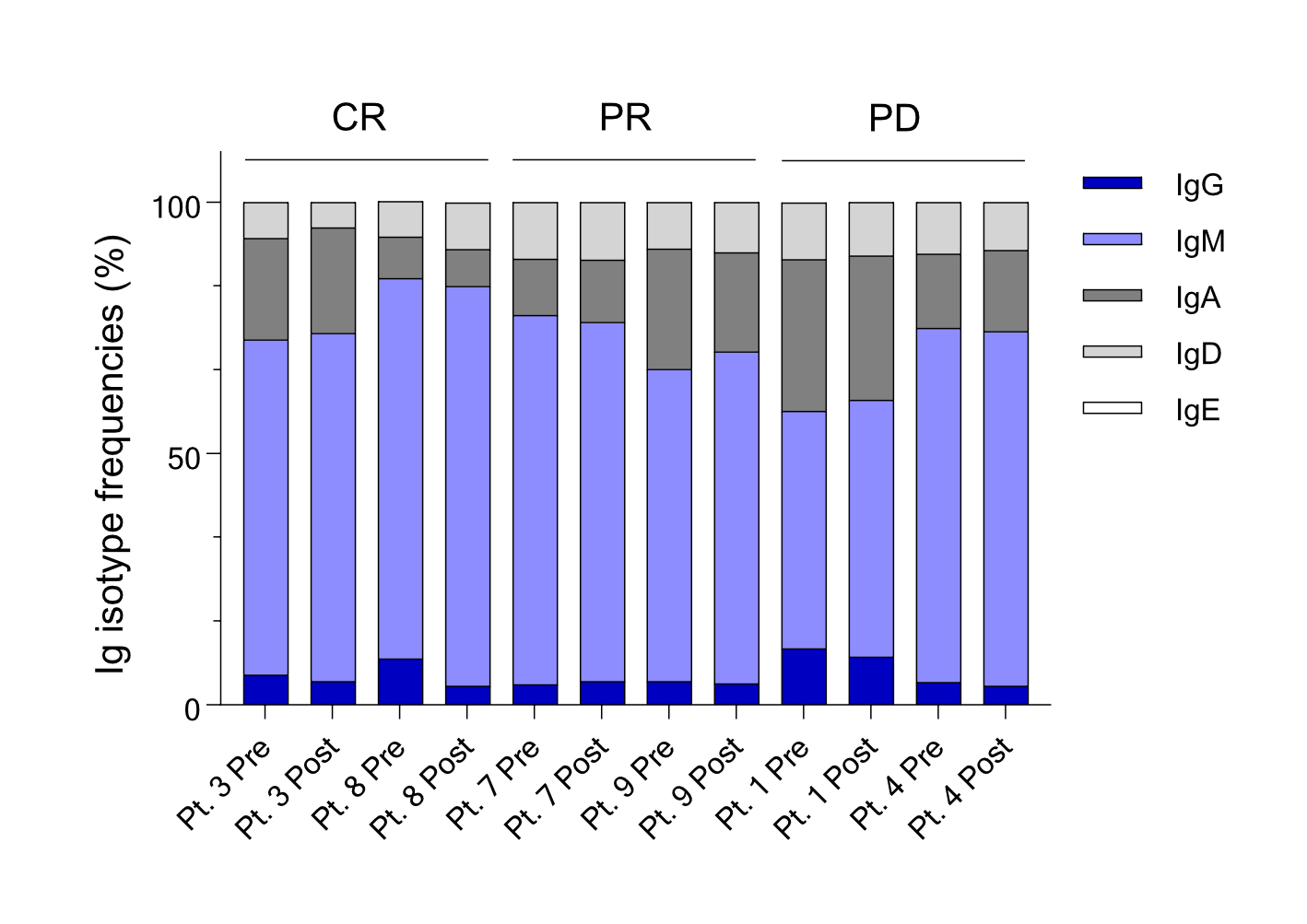

Supplement: Supplementary Figure 2 — Memory B cell immunoglobulin isotype analysis. Immunoglobulin isotype distribution (IgG, IgM, IgD, IgA and IgE) in the memory B cell fraction obtained from IgH CDR3 sequencing of melanoma patients, pre- and post-immunotherapy, represented as the average percentage of the two replicates. PD, progressive disease; PR, partial response; CR, complete response. [file Image2.jpeg]

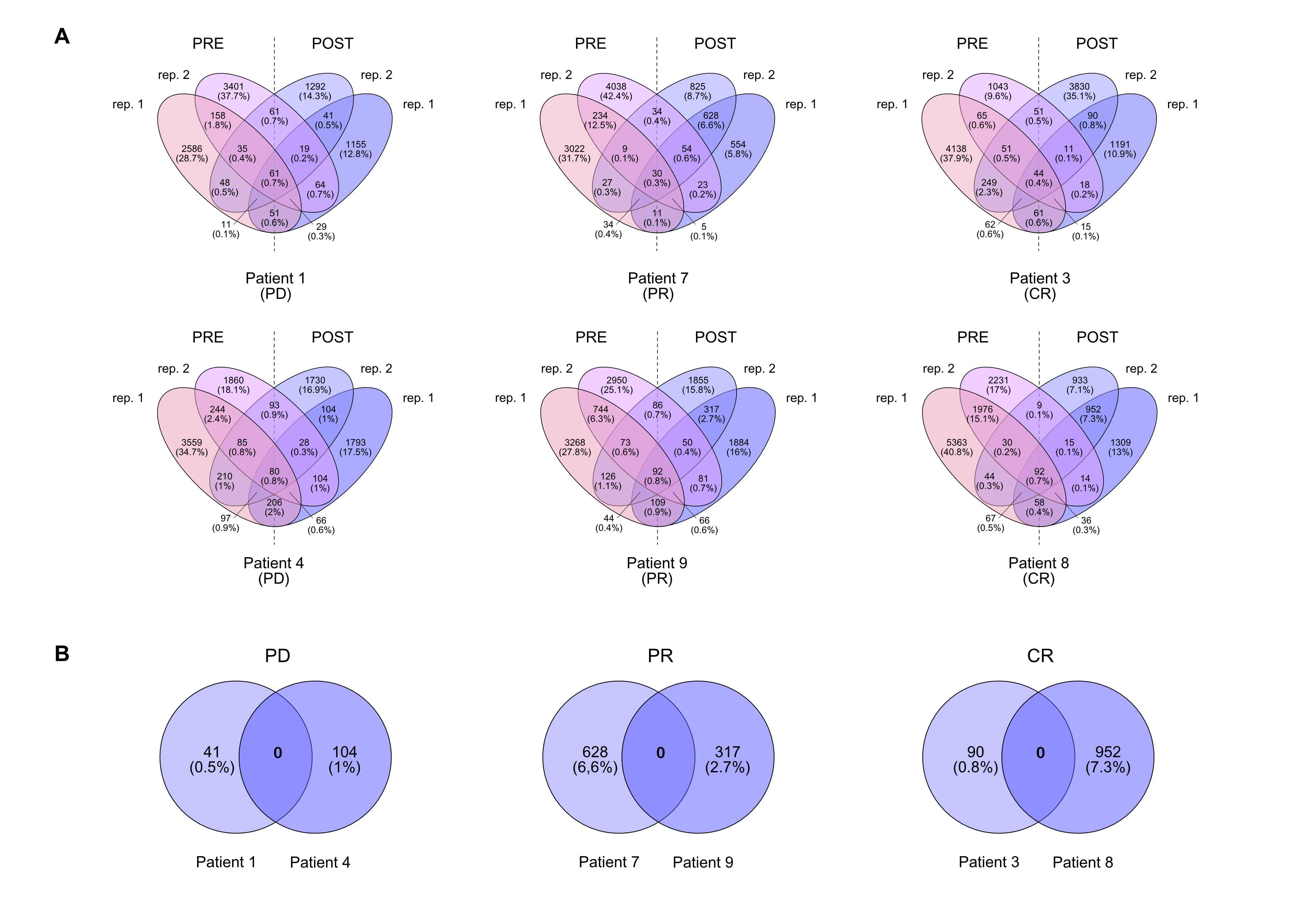

Supplement: Supplementary Figure 3 — Comparison of CDR3 sequences between melanoma patients following the IgH CDR3 sequencing. (A) Venn diagrams showing CDR3 sequences overlap between the two replicates pre- (PRE) and post-treatment (POST) with nivolumab, from IgH CDR3 sequencing. (B) CDR3 sequences were matched based on the clinical response for each individual patient, considering only those sequences found post-treatment and overlapping the two sequencing replicates. Percentages in parentheses represent the fraction of CDR3 counts relative to the total count for each patient. BCR, B-cell receptor; CDR3, complementarity-determining region 3; PD, progressive disease; PR, partial response; CR, complete response. [file Image3.jpeg]

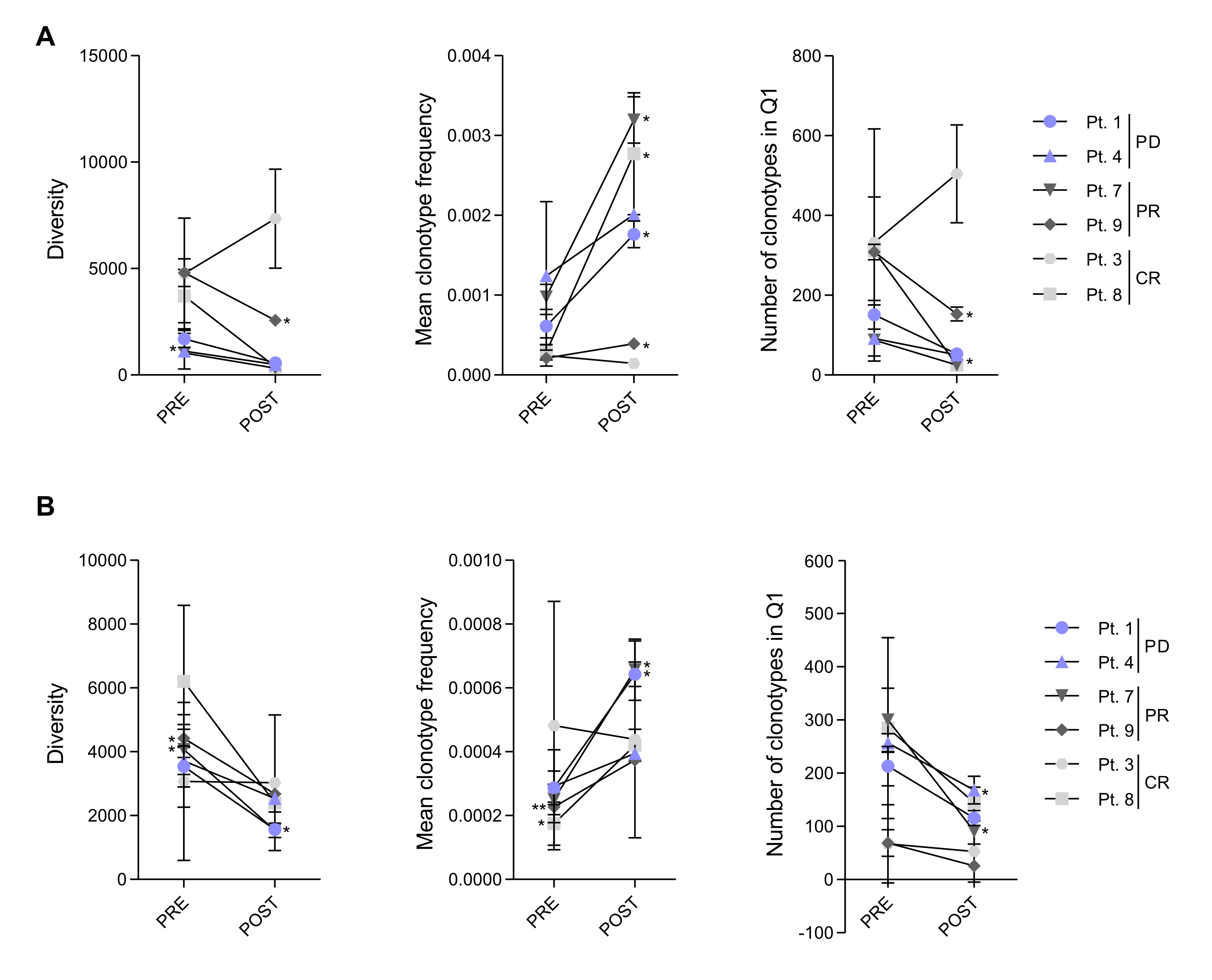

Supplement: Supplementary Figure 4 — Diversity, mean frequency, and Q1 enrichment from CDR3 IgG and IgH sequencing. Total clonotype diversity, mean clonotype frequency, and number of clonotypes in the upper 20% quantile (Q1) were represented as the average +SD of the two replicates for the IgG CDR3 (A) and IgH CDR3 (B) sequencing, comparing pre- (PRE) and post-treatment (POST) with nivolumab. PD, progressive disease; PR, partial response; CR, complete response. [file Image4.jpeg]

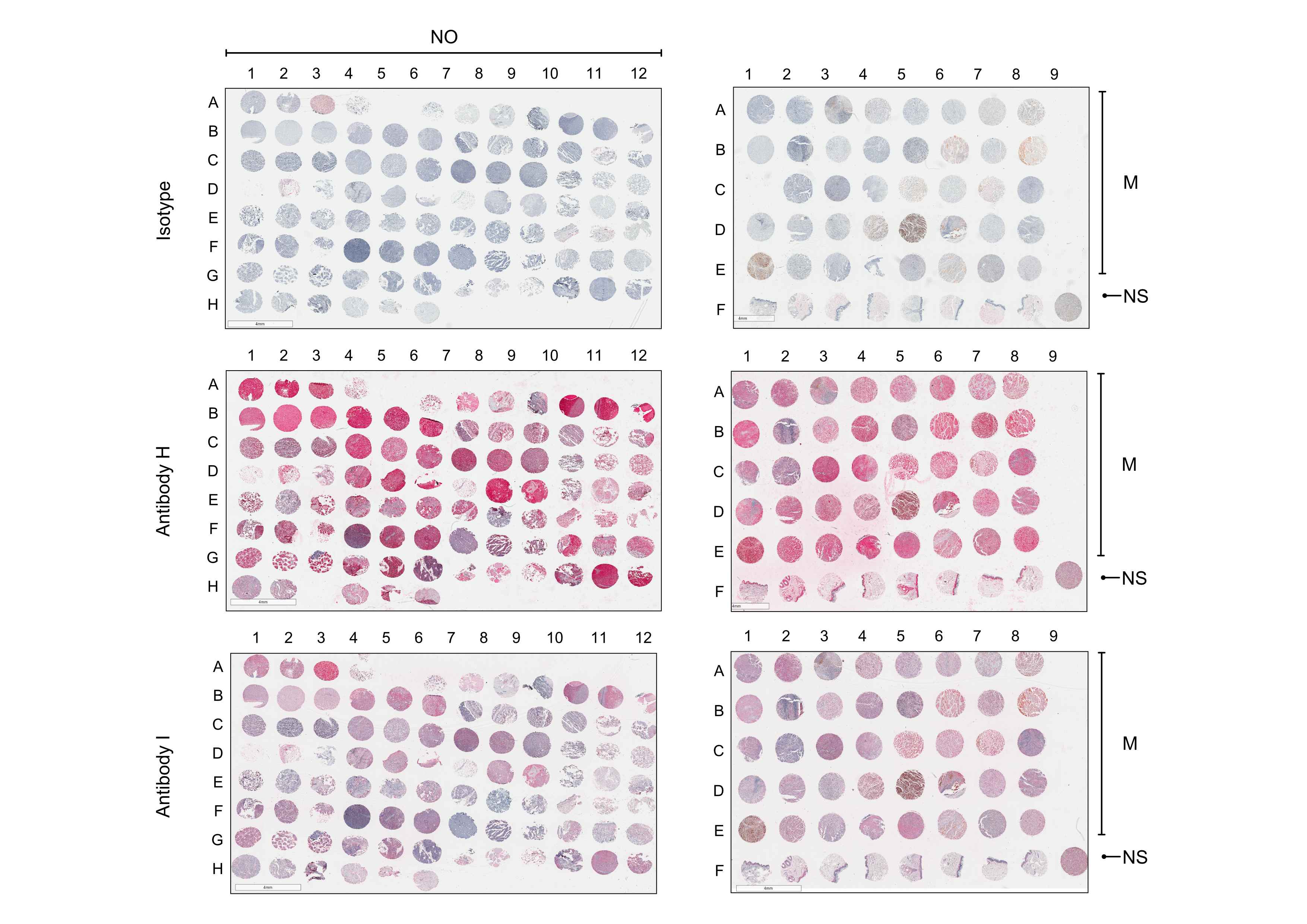

Supplement: Supplementary Figure 5 — Antibody testing on tissue microarray. Complete panel of the tissue microarray (TMA) of normal organs (NO), melanoma (M) and normal skin (NS) samples, stained with either antibody H or I, and isotype control antibody. Related to Supplementary Table S3 . [file Image5.jpeg]
