# Supplementary material for: Circulating memory B-cell receptor repertoire analysis identifies novel candidate antibodies against metastatic melanoma in immunotherapy-responsive patients
Source: Front Immunol. 2025 Oct 9;16:1636722. doi: 10.3389/fimmu.2025.1636722 (PMC12548061; doi:10.3389/fimmu.2025.1636722)
Supplement: Supplementary file 6 [file Table1.docx]

**Supplementary Table 1. Statistical analysis of IgG CDR3 BCR sequencing.** Patient samples (1 to 9) are ordered based on clinical response (PD, progressive disease; PR, partial response; CR, complete response). The total number of sequence reads is reported for both sequencing replicates (rep 1 and rep 2), pre- and post-treatment. Clone diversity represents the number of unique CDR3 sequences. Mean clonotype frequency indicates the average relative abundance of unique CDR3 sequences. BCR, B-cell receptor; CDR3, complementarity-determining region 3.

| **Sample** | **Clinical response** | **Reads** | **Diversity** | **Mean clonotype frequency** | **Mean CDR3 nucleotide length** |
| --- | --- | --- | --- | --- | --- |
| 1-pre-rep1-IGG | PD | 4235 | 1400 | 7.14e-4 | 52,03 |
| 1-pre-rep2-IGG | PD | 6376 | 1977 | 5.06e-4 | 51,21 |
| 1-post-rep2-IGG | PD | 2423 | 609 | 1.64e-3 | 51,10 |
| 1-post-rep1-IGG | PD | 1635 | 532 | 1.88e-3 | 51,01 |
| 4-pre-rep2-IGG | PD | 2054 | 527 | 1.90e-3 | 53,52 |
| 4-pre-rep1-IGG | PD | 5728 | 1711 | 5.84e-4 | 53,10 |
| 4-post-rep1-IGG | PD | 2083 | 503 | 1.99e-3 | 54,06 |
| 4-post-rep2-IGG | PD | 2123 | 492 | 2.03e-3 | 54,39 |
| 7-pre-rep1-IGG | PR | 2777 | 917 | 1.09e-3 | 52,04 |
| 7-pre-rep2-IGG | PR | 3363 | 1155 | 8.66e-4 | 52,23 |
| 7-post-rep1-IGG | PR | 886 | 334 | 2.99e-3 | 52,26 |
| 7-post-rep2-IGG | PR | 771 | 294 | 3.40e-3 | 52,31 |
| 9-pre-rep1-IGG | PR | 10287 | 4347 | 2.30e-4 | 52,27 |
| 9-pre-rep2-IGG | PR | 11839 | 5266 | 1.90e-4 | 52,04 |
| 9-post-rep1-IGG | PR | 5100 | 2397 | 4.17e-4 | 52,15 |
| 9-post-rep2-IGG | PR | 5947 | 2732 | 3.66e-4 | 52,25 |
| 8-pre-rep1-IGG | CR | 17252 | 4592 | 2.18e-4 | 50,05 |
| 8-pre-rep2-IGG | CR | 9234 | 2825 | 3.54e-4 | 49,26 |
| 8-post-rep1-IGG | CR | 699 | 302 | 3.31e-3 | 51,55 |
| 8-post-rep2-IGG | CR | 1017 | 449 | 2.23e-3 | 52,14 |
| 3-pre-rep1-IGG | CR | 17831 | 6615 | 1.51e-4 | 52,49 |
| 3-pre-rep2-IGG | CR | 5472 | 2933 | 3.41e-4 | 52,21 |
| 3-post-rep1-IGG | CR | 21411 | 8988 | 1.11e-4 | 52,42 |
| 3-post-rep2-IGG | CR | 14526 | 5688 | 1.76e-4 | 52,43 |

**Supplementary Table 2. Statistical analysis of IgH CDR3 BCR sequencing.** Patient samples (1 to 9) are ordered based on clinical response (PD, progressive disease; PR, partial response; CR, complete response). The total number of sequence reads is reported for both sequencing replicates (rep 1 and rep 2), pre- and post-treatment. Clone diversity represents the number of unique CDR3 sequences. Mean clonotype frequency indicates the average relative abundance of unique CDR3 sequences. BCR, B-cell receptor; CDR3, complementarity-determining region 3.

| **Sample** | **Clinical response** | **Reads** | **Diversity** | **Mean clonotype frequency** | **Mean CDR3 nucleotide length** |
| --- | --- | --- | --- | --- | --- |
| 1-pre-rep1-IGH | PD | 7856 | 3085 | 3,24E-04 | 47,66 |
| 1-pre-rep2-IGH | PD | 10122 | 3998 | 2,50E-04 | 47,39 |
| 1-post-rep1-IGH | PD | 5002 | 1626 | 6,15E-04 | 48,2 |
| 1-post-rep2-IGH | PD | 5381 | 1492 | 6,70E-04 | 46,71 |
| 4-pre-rep1-IGH | PD | 10823 | 4738 | 2,11E-04 | 46,64 |
| 4-pre-rep2-IGH | PD | 8870 | 2684 | 3,73E-04 | 47,02 |
| 4-post-rep1-IGH | PD | 5745 | 2501 | 4,00E-04 | 46,23 |
| 4-post-rep2-IGH | PD | 7162 | 2573 | 3,89E-04 | 47,74 |
| 7-pre-rep1-IGH | PR | 9497 | 3515 | 2,84E-04 | 46,8 |
| 7-pre-rep2-IGH | PR | 13503 | 4631 | 2,16E-04 | 46,3 |
| 7-post-rep1-IGH | PR | 4316 | 1697 | 5,89E-04 | 47 |
| 7-post-rep2-IGH | PR | 3100 | 1379 | 7,25E-04 | 47,02 |
| 9-pre-rep1-IGH | PR | 6729 | 4618 | 2,17E-04 | 48,34 |
| 9-pre-rep2-IGH | PR | 5735 | 4219 | 2,37E-04 | 48,78 |
| 9-post-rep1-IGH | PR | 3375 | 2677 | 3,74E-04 | 48,54 |
| 9-post-rep2-IGH | PR | 3401 | 2675 | 3,74E-04 | 48,11 |
| 3-pre-rep1-IGH | CR | 7603 | 4821 | 2,07E-04 | 46,78 |
| 3-pre-rep2-IGH | CR | 1696 | 1321 | 7,57E-04 | 45,16 |
| 3-post-rep1-IGH | CR | 6863 | 4532 | 2,21E-04 | 46,23 |
| 3-post-rep2-IGH | CR | 1865 | 1521 | 6,57E-04 | 46,46 |
| 8-pre-rep1-IGH | CR | 17875 | 7888 | 1,27E-04 | 46,45 |
| 8-pre-rep2-IGH | CR | 8773 | 4512 | 2,22E-04 | 46,01 |
| 8-post-rep1-IGH | CR | 4710 | 2194 | 4,56E-04 | 47,33 |
| 8-post-rep2-IGH | CR | 6035 | 2599 | 3,85E-04 | 47,29 |

**Supplementary Table 3. Tissue microarray analysis.** Immunological evaluation of antibody H and I binding on a panel of melanoma, normal skin, and normal organ tissue microarray (TMA) samples.

**Supplementary Table 4. Analysis of antibody I and H staining on tissue microarray.** Statistical difference (P-values < 0.05) between antibody staining on melanoma samples versus normal skin and normal tissue samples was assessed by Fisher's exact test based on the binding intensity score: 0, negative (N); 0.5, very weak (VW); 1, weak (W); 2, intermediate (I); 2.5, intermediate-strong (IS); 3, strong (S). Antibody binding positivity was determined by examining signals that fell within the range of weak to strong (W + I + IS + S), and then compared for significance against negative and very weak staining scores combined (N + VW). Referred to Figure 5.

|  | **N + VW** | **W + I + IS + S** | Total |
| --- | --- | --- | --- |
| **Melanoma Ab I** | 7 | 34 | 41 |
| **Normal tissue Ab I** | 44 | 39 | 83 |
| Total | 51 | 73 | 124 |
|  |  |  |  |
| *P value* | *0,0003* |  |  |
|  |  |  |  |
|  | **N + VW** | **W + I + IS + S** | Total |
| **Melanoma Ab I** | 7 | 34 | 41 |
| **Normal skin Ab I** | 3 | 8 | 11 |
| Total | 10 | 42 | 52 |
|  |  |  |  |
| *P value* | *0,42* |  |  |
|  |  |  |  |
|  | **N + VW** | **W + I + IS + S** | Total |
| **Melanoma Ab H** | 3 | 38 | 41 |
| **Normal tissue Ab H** | 3 | 84 | 87 |
| Total | 6 | 122 | 128 |
|  |  |  |  |
| *P value* | *0,36* |  |  |
|  |  |  |  |
|  | **N + VW** | **W + I + IS + S** | Total |
| **Melanoma Ab H** | 3 | 38 | 41 |
| **Normal skin Ab H** | 3 | 8 | 11 |
| Total | 3 | 46 | 49 |
|  |  |  |  |
| *P value* | *1* |  |  |
